# Liver transcriptome profile in pigs with extreme phenotypes of intramuscular fatty acid composition

**DOI:** 10.1186/1471-2164-13-547

**Published:** 2012-10-11

**Authors:** Yuliaxis Ramayo-Caldas, Nuria Mach, Anna Esteve-Codina, Jordi Corominas, Anna Castelló, Maria Ballester, Jordi Estellé, Noelia Ibáñez-Escriche, Ana I Fernández, Miguel Pérez-Enciso, Josep M Folch

**Affiliations:** 1 Centre de Recerca en Agrigenòmica (CRAG), Consorci CSIC-IRTA-UAB-UB, Campus UAB, Bellaterra, 08193, Spain; 2 INRA, UMR 1313 Génétique Animale et Biologie Intégrative (GABI), Equipe Génétique Immunité Santé, Jouy-en-Josas, F-78352, France; 3 AgroParisTech, UMR 1313 GABI, Jouy-en-Josas, F-78352, France; 4 CEA, DSV/iRCM/SREIT/LREG, Jouy-en-Josas, F-78352, France; 5 Genètica i Millora Animal, IRTA Lleida, Lleida, 25198, Spain; 6 Departamento de Mejora Genética Animal, INIA, Ctra. De la Coruña km. 7, Madrid, 28040, Spain; 7 Institut Català de Recerca i Estudis Avançats (ICREA), Barcelona, Spain; 8 Departament de Ciència Animal i dels Aliments, Facultat de Veterinària, Universitat Autònoma de Barcelona, 08193, Bellaterra, Spain

## Abstract

**Background:**

New advances in high-throughput technologies have allowed for the massive analysis of genomic data, providing new opportunities for the characterization of the transcriptome architectures. Recent studies in pigs have employed RNA-Seq to explore the transcriptome of different tissues in a reduced number of animals. The main goal of this study was the identification of differentially-expressed genes in the liver of Iberian x Landrace crossbred pigs showing extreme phenotypes for intramuscular fatty acid composition using RNA-Seq.

**Results:**

The liver transcriptomes of two female groups (H and L) with phenotypically extreme intramuscular fatty acid composition were sequenced using RNA-Seq. A total of 146 and 180 unannotated protein-coding genes were identified in intergenic regions for the L and H groups, respectively. In addition, a range of 5.8 to 7.3% of repetitive elements was found, with SINEs being the most abundant elements. The expression in liver of 186 (L) and 270 (H) lncRNAs was also detected. The higher reproducibility of the RNA-Seq data was validated by RT-qPCR and porcine expression microarrays, therefore showing a strong correlation between RT-qPCR and RNA-Seq data (ranking from 0.79 to 0.96), as well as between microarrays and RNA-Seq (r=0.72). A differential expression analysis between H and L animals identified 55 genes differentially-expressed between groups. Pathways analysis revealed that these genes belong to biological functions, canonical pathways and three gene networks related to lipid and fatty acid metabolism. In concordance with the phenotypic classification, the pathways analysis inferred that linolenic and arachidonic acids metabolism was altered between extreme individuals. In addition, a connection was observed among the top three networks, hence suggesting that these genes are interconnected and play an important role in lipid and fatty acid metabolism.

**Conclusions:**

In the present study RNA-Seq was used as a tool to explore the liver transcriptome of pigs with extreme phenotypes for intramuscular fatty acid composition. The differential gene expression analysis showed potential gene networks which affect lipid and fatty acid metabolism. These results may help in the design of selection strategies to improve the sensorial and nutritional quality of pork meat.

## Background

Pigs, an important source of human food, accounting for over 40% of the meat produced worldwide. In addition, due to the similarities in anatomy and physiology with humans, they have been used in biomedicine as an important animal model for the study of the genetic basis of metabolic diseases such as obesity, type II diabetes, metabolic syndrome and atherosclerosis. As well it is often mentioned as the preferred animal species for organ xenotransplantation [1,2].

Over the last decade, a growing awareness of the association between diet and health has led nutritional quality to become a relevant factor in consumers’ food choices. A major development has been the recognition that certain fatty acids (FA), such as oleic acid, and α-linolenic acid (ALA), can improve human health status and prevent disease [3,4]. Production of meat with a fatty acids profile more in line with public health recommendations has the potential to improve long-term human health without requiring substantial changes in consumer habits. It is well known that the fatty acid meat composition of pigs is largely dependent on genotype, physiological status and environmental factors such as nutrition [5-11].

The liver a highly specialized organ present in vertebrates and other animals regulates a wide variety of metabolic processes, which play a key role in the digestive function, the decomposition of red blood cells, hormone production and detoxification. Together with adipose tissue and skeletal muscle, the liver is crucial in regulating lipid metabolism. In pigs, the liver is the primary site of *de novo* cholesterol synthesis and fatty acid oxidation, whereas lipogenesis occurs essentially in liver and adipose tissues [12-16].

In the last few years, new high-throughput technologies have been developed for the massive analysis of genomic data. These methodologies yield new opportunities to explore the genetic variability of populations, as well as the characterization of the transcriptome architectures. Until the development of Next-generation sequencing (NGS) technologies, most mRNA expression studies have used microarray or quantitative PCR-based (qPCR) approaches. The development of RNA-Seq, a method based on NGS which consisting of the direct sequencing of RNA molecules present in a given sample, has provided a new tool for both transcriptome characterization and gene expression profiling. In RNA-Seq, the counts corresponding to each transcript can be used for quantification and these sequences can be mapped to the genome for their annotation. In comparison to microarrays, RNA-Seq provides a higher dynamic range, specificity and sensibility [17]. In addition, it provides a picture of the transcriptome, allowing the characterization of alternative splicing, variation in the usage of promoters and polyadenilation sites, non-coding RNAs (ncRNA), single nucleotide variants (SNVs) and transposable elements. Furthermore, RNA-Seq data may allow the discovery of novel transcripts and long intergenic non-coding RNAs (lncRNAs) [18-20].

Recent studies in livestock species have employed RNA-Seq to explore the transcriptome of animal products, such as cow milk [21], bovine embryos [22], and tissue as pig gonads [23], liver, muscle, and abdominal fat [24], sheep bone [25] and bovine abomasal [26]. However, most of the RNA-Seq studies in pigs have included analysis of only a few animals, and ignored within group intrinsic variability. For instance, two single animals of different breeds were compared by Esteve-Codina *et al*., (2011) and three tissues in two phenotypically extreme full-sib F_2_ females formed the basis of Chen *et al*., (2011) study.

The main goal of this study was the identification of differentially-expressed genes in the liver of groups of Iberian x Landrace crossbred pigs showing extreme phenotypes for intramuscular fatty acid composition using RNA-Seq. In addition, the porcine hepatic transcriptome was analyzed and transposable elements, new putative protein-coding genes and lncRNAs were identified.

## Results and discussion

### Phenotypic variation between extreme groups

**Figure 1:**
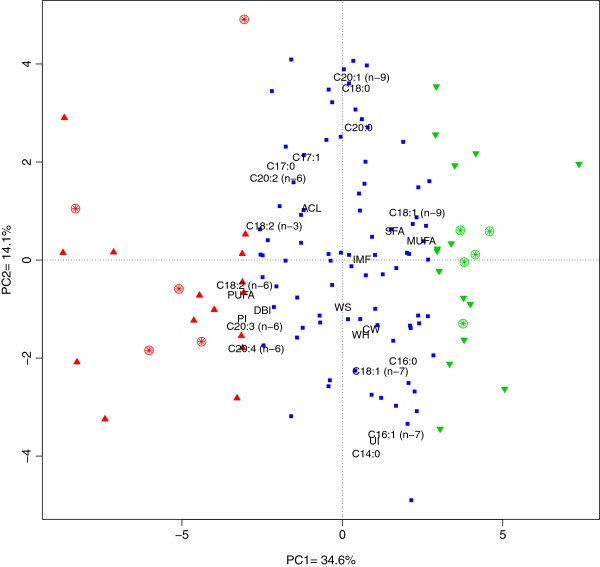
**Graphical representation of the first and second principal components summarizing the phenotype variation of traits related to carcass quality and intramuscular fatty acid composition.** Low group (L) animals are indicated with green triangles and High (H) animals with red triangles, while blue squares represent the whole populations. The ten sequenced animals are represented with circles containing asterisks. Abbreviations are defined in Table 1.

Analyzed animals were a backcross (25% Iberian x 75% Landrace) obtained by crossing three Iberian (Guadyerbas) boars and 30 Landrace sows. Subsequently five F1 boards were backcrossed with 26 Landrace sows. A Principal Component Analysis (PCA) was performed to obtain the low-dimensional representation of the data and to describe the phenotypic variation of traits related to carcass quality and intramuscular fatty acid composition. The first two principal components explained the 48.7% of the global phenotypic variance of these traits (PC1=34.6%, PC2=14.1%, Figure 1).

According to the score information for the first principal component the animals were ranked in two groups High (H) and Low (L), of 20 individuals each. Figure 1 shows two clusters of animals with the relative weight of all traits in the two first principal components. The first principal component grouped several traits related to the profile of fatty acids in *Longissimus dorsi* (LD) muscle. Group L showed a higher proportion of saturated (SFA) and monounsaturated fatty acids (MUFA), including palmitoleic and oleic acids. Conversely, H group had a higher content of polyunsaturated acids (PUFA) and related indices like the double bond index (DBI), the unsaturated index (UI) and the peroxidability index (PI). Remarkably, H group also presented a higher proportion of essential PUFA, like linolenic (LA), ALA, eicosadienoic (EDA), eicosatrienoic (ETE) and arachidonic (AA) acids (Table 1). These phenotypic differences are likely determined by genetic variability in: 1) absorption of LA and ALA acids; 2) elongation and desaturation of essential PUFA to longer-chain ω−3 and ω−6 fatty acids; 3) *de novo* synthesis and metabolism of palmitoleic and oleic acids; and 4) transport deposition, storage or degradation and oxidation of all these fatty acids.

**Table 1:** Mean comparison ± standard deviation between high and low groups for the traits included in the principal components analysis (PCA)

**Carcass quality**	**Mean Low**	**Mean High**	**Significance**
Carcass height (CH)	72.79 ± 9.90	69.57 ± 12.73	NS
Weight of ham (WH)	19.88 ± 2.46	19.66 ± 3.10	NS
Weight of Shoulder (WS)	10.26 ± 1.25	10.93 ± 2.10	NS
Intramuscular fat (IMF)	2.21 ± 0.88	1.49 ± 0.38	NS
**Fatty acids**			
***Saturated FA***			
Myristic acid (C14:0)	1.22 ± 0.13	1.12 ± 0.12	NS
Palmitic acid (C16:0)	23.78 ± 0.79	21.39 ± 0.69	***
Heptadecanoic acid (C17:0)	0.20 ± 0.03	0.33 ± 0.09	***
Stearic acid (C18:0)	14.65 ± 1.16	13.69 ± 0.77	*
Arachidic acid (C20:0)	0.26 ± 0.05	0.21 ± 0.08	NS
***Monounsaturated FA***			
Palmitoleic acid (C16:1 n-7)	2.74 ± 0.24	2.20 ± 0.33	***
Heptadecenoic acid (C17:1)	0.20 ± 0.05	0.32 ± 0.20	**
Oleic acid (C18:1 n-9)	42.57 ± 1.34	34.92 ± 2.96	***
Octadecenoic acid (C18:1 n-9)	4.04 ± 0.27	3.70 ± 0.31	*
Eicosenoic acid (C20:1 n-9)	0.86 ± 0.08	0.77 ± 0.07	NS
***Polyunsaturated FA***			
Linoleic acid (C18:2 n-6)	7.16 ± 0.52	15.11 ± 1.65	***
α-Linolenic acid (C18:2 n-3)	0.46 ± 0.08	1.10 ± 0.56	***
Eicosadienoic acid (C20:2 n-6)	0.41 ± 0.05	0.63 ± 0.13	***
Eicosatrienoic acid (C20:3 n-6)	0.16 ± 0.03	0.53 ± 0.15	***
Arachidonic acid (C20:4 n-6)	0.84 ± 0.17	3.10 ± 0.77	***
***Metabolic ratios***			
Average Chain Length (ACL)	17.44 ± 0.02	17.49 ± 0.12	*
Saturated FA (SFA)	40.12 ± 1.51	36.72 ± 1.22	***
Monounsaturated FA (MUFA)	50.72 ± 1.56	42.38 ± 3.13	***
Polyunsaturated FA (PUFA)	9.03 ± 0.64	20.46 ± 2.21	***
Peroxidability index (PI)	12.28 ± 3.80	32.43 ± 4.43	***
Double-bond index (DBI)	0.65 ± 0.20	0.91 ± 0.03	***
Unsaturated index (UI)	1.63 ± 0.50	2.48 ± 0.10	***

NS: p-value > 0.05, * p-value < 0.05, ** p-value < 0.01, *** p-value < 0.001.

Previous studies have reported that, in both backfat and LD muscle, Iberian pigs have higher percentages of palmitic acid, oleic acid, SFA and MUFA, and lower concentrations of LA and ALA acids than commercial breeds [7,27,28]. Moreover, Pascual *et al*., (2007) [9] reported that Landrace pigs have a higher content of LA and AA acids in their muscle than other commercial breeds. In general, fatter pigs show higher proportions of SFA and MUFA, but less PUFA than lean pigs [6,29]. The genetic architecture of intramuscular FA composition in the Iberian x Landrace backcross was described in a genome-wide association study (GWAS), showing 43 chromosomal intervals associated with these traits [30]. Since all animals were raised and fed under the same standard management conditions, differences between H and L groups are probably caused by the segregation within the analyzed animals of Iberian and Landrace alleles.

Phenotypic means between groups were compared and significant statistical differences in 73% of the analyzed traits was noted (19/26), mainly relating to intramuscular fatty acid composition (Table 1). The maximum differences between groups were observed for the profiles of essential PUFAs (AA, ETE, LA and ALA acids). Significant differences were also observed for PI and the percentage of palmitic, palmitoleic, heptadecenoic and heptadecanoic acids. From the 20 extreme animals, 10 females were selected for RNA sequencing (five per group). Pedigree information was used to select animals representing the parental genetic diversity. In addition, full-sibs within groups were avoided, animals within groups had different mothers, and four different fathers were selected per group. However, interesting familial relationships between animals of different groups were retained: there were two pairs of full-sibs and two pair of maternal half-sibs belonging to opposite groups. As before, the phenotypic means differed between groups. However, due to the reduced sample size, only sixteen traits showed significant differences (Additional file 1, Table S1).

### Mapping and annotation

The pig liver transcriptome of two groups (H=5, L=5) of phenotypically extreme females for intramuscular fatty acid composition was sequenced. After removal of sequencing adaptors and low-complexity reads, Tophat software was employed [31] to map the reads against the reference pig genome assembly Sscrofa 9.61. A total of 136.65 M of 100 bp single-end reads (7.28 – 12.43 M of single-end reads per individual) were obtained from two lanes of an *Illumina Hi-Seq 2000* machine. Observed percentages of mapped reads per individual were higher (around 71.42 – 77.75%) than obtained previously in other porcine transcriptome studies; 61.4 - 65.6% [24] and 66.7 [23]. The number of reads and the mean percentages of mapped reads were equivalent for the H and L groups (Table 2).

**Table 2:** Number of single-end 100 bp reads obtained and percentages of mapped reads per animal

**Animal**^**1**^	**Total M**^**2**^	**Mapped M**^**2**^	**%**
L1	9.87	7.28	73.82
L2	12.44	8.88	71.42
L3	13.98	10.62	75.95
L4	11.15	8.47	75.96
L5	14.42	11.21	77.75
H1	16.05	12.43	77.45
H2	12.57	9.77	77.68
H3	14	10.75	76.77
H4	14.94	11.52	77.14
H5	17.23	13.1	76.03
**Total**	136.65	104.03	76.13

^1^ L1 to L5 and H1 to H5 correspond to animals of the L and H groups, respectively.

^2^ Indicate millions of reads.

S-MART [32] was used to calculate the proportion of reads mapping to exons, introns and 1kb upstream/downstream of the annotated genes. As expected, the highest percentage of reads mapped to exons (60.4 – 66.5%), while 11.1 – 16.4% corresponded to introns and the lowest percentage was located either 1 kb upstream or downstream of the annotated genes (4.06 – 5.46%) (Table 3). The proportion of reads mapped to exons of annotated genes was in accordance with the study of Chen *et al*., (2011) in three pig tissues (60.2 – 74.9%), but was higher than that reported by Esteve-Codina *et al*., 2011 (44.1%) in porcine male gonads. These differences (≈ 12%) may be explained by the use of different versions of both the pig genome assembly and annotation, Sscrofa9.61 in the present study and Sscrofa9.58 in Esteve-Codina *et al*., (2011). Moreover, in the present study a newer version of Tophat was used, which includes improvements in mapping. However, differences between tissues in the proportion of annotated genes cannot be ruled out.

**Table 3:** Proportion of reads mapping to exons, introns or within 1 Kb upstream or downstream of the annotated genes

**Animal**^**1**^	**% Exons**	**% Introns**	**% _5’ or _3’**^**2**^
L1	60.44	16.44	5.03
L2	66.48	13.59	4.06
L3	65.52	13.06	5.31
L4	63.35	14.71	4.57
L5	62.86	12.71	4.6
H1	63.54	13.52	5.44
H2	62.1	15.08	4.78
H3	64.87	14.11	4.28
H4	65.95	11.12	5.46
H5	66.46	12.98	4.44

^1^ L1 to L5 and H1 to H5 correspond to animals of the L and H groups, respectively.

^2^ Reads located either 1 Kb upstream or downstream of the annotated genes.

The total number of assembled transcripts with cufflinks was in agreement with the previously reported pig liver transcriptome [24]. These transcripts fall into the following categories: annotated exons (8.7 – 11%), intron retention events (11 – 13.5%), intergenic transcripts (19.1 – 21.7%), potentially novel isoforms of genes (17.1 – 20.3%), known isoforms (14.7 – 17.8%), pre-mRNA molecules (2 – 3.3%) and polymerase run-on fragments (5.9 – 8.3%) (Table 4).

**Table 4:** Number of transcripts assembled (TA) with Cufflinks and the percentage they represent in each sample

**Code**	**L1**		**L2**		**L3**		**L4**		**L5**		**H1**		**H2**		**H3**		**H4**		**H5**	
	***TA***	***%***	***TA***	***%***	***TA***	***%***	***TA***	***%***	***TA***	***%***	***TA***	***%***	***TA***	***%***	***TA***	***%***	***TA***	***%***	***TA***	***%***
=	1971	8.7	2486	10.4	2526	11	2244	9.2	2762	10.2	2642	10.1	2363	9.4	2761	9.9	2119	10.4	2838	10.5
c	4059	17.8	4202	17.6	3953	17.2	4203	17.3	4289	15.9	3955	15.1	4083	16.3	4183	15.0	3569	17.5	3961	14.7
e	745	3.3	508	2.1	518	2.3	672	2.8	576	2.1	616	2.4	736	2.9	604	2.2	489	2.4	529	2.0
i	3115	13.7	2719	11.4	2629	11.4	3153	13.0	3248	12.0	3301	12.6	3373	13.5	3637	13.1	2250	11.0	3234	12.0
j	4348	19.1	4709	19.7	4436	19.3	4666	19.2	5373	19.9	5288	20.2	4833	19.3	5658	20.3	3488	17.1	5428	20.1
o	1067	4.7	1175	4.9	1139	5.0	1157	4.8	1237	4.6	1142	4.4	1154	4.6	1170	4.2	1129	5.5	1204	4.5
p	1570	6.9	1656	6.9	1566	6.8	1682	6.9	1635	6.1	1603	6.1	1606	6.4	1676	6.0	1699	8.3	1598	5.9
s	60	0.3	77	0.3	72	0.3	76	0.3	94	0.4	86	0.3	71	0.3	72	0.3	68	0.3	100	0.4
u	4447	19.5	4560	19.1	4541	19.7	4789	19.7	5468	20.2	5456	20.9	5089	20.3	5680	20.4	4421	21.7	5638	20.9
x	1392	6.1	1838	7.7	1630	7.1	1663	6.8	2346	8.7	2066	7.9	1761	7.0	2415	8.7	1192	5.8	2496	9.2
**Total**	22774	100	23930	100	23010	100	24305	100	27028	100	26155	100	25069	100	27856	100	20424	100	27026	100

Class codes described by Cuffcompare: "=" Exactly equal to the reference annotation, "c " Contained in the reference annotation, "e" possible pre-mRNA molecule, "i " An exon falling into an intron of the reference, "j " New isoforms, "o" Unknown, generic overlap with reference, "p" Possible polymerase run-on fragment, “s” An intron of the transfrag overlapping a reference intron on the opposite strand, "u" Unknown, intergenic transcript, “x” Exonic overlap with reference on the opposite strand. L1 to L5 and H1 to H5 correspond to animals of the L and H groups, respectively.

### Gene expression analysis

The total amount of expressed genes in liver was similar between groups (L= 8797 – 10161, H= 8765 – 10083). Taking into account only those genes with a mean FPKM (normalized number of fragments per kilobase of exon per million reads) higher than zero, an aggregate of 10,485 expressed genes in L and 10,626 in H groups was observed. A total of 10,280 common genes were expressed in both groups. The correlation of mean gene expression levels between both groups (H vs L) was very high (r = 0.99), suggesting that the major fraction of the liver transcriptome is conserved between groups. Gene expression distribution reveals that less than 10% of these genes were expressed between 1 – 10 FPKM; around 42% between 10 FPKM - 100 FPKM; 38% among 100 – 1000 FPKM and, approximately, 8% more than 1000 FPKM (Additional file 2, Figure S1).

All 10 individuals were also assayed with the GeneChip® Porcine microarray (*Affymetrix,* Santa Clara, CA) which allows the expression analysis of 20,201 *Sus scrofa* genes. After probe normalization, correlation between the expression data of microarrays and RNA-Seq was calculated. In accordance with previous studies [17,23], a strong Spearman correlation (r=0.72) was observed (Additional file 3, Figure S2). Results from both technologies were, in general, more similar for genes that showed intermediate expression values, whereas major differences were observed for low and high expressed genes in the *Affymetrix* microarray data. This pattern can be explained by the higher dynamic range of RNA-Seq [17,33]. Finally, in line with the previous description of liver transcriptome [34], the top 100 expressed genes showed an overrepresentation in biological gene ontologies related to oxidoreductase activity, transport, proteolysis, translation, signal transduction, cholesterol homeostasis and lipid transport (p < 0.001).

### Transposable elements analysis

The percentage of repetitive elements identified in the pig liver transcriptome was around5.8 – 7.3% (Additional file 4, Table S2), similar to that found in male gonad transcriptome (7.3%) [23]. However, it should be noted that the total length of base pairs masked and the total number of transcripts were higher in male gonad transcriptome [23] than in our study. Two possible explanations may account for these differences: 1) in liver from 7.3 – 12.4 M of single-end reads per individual were obtained (Table 2), whereas in gonads [23] a total of 20 M paired-end reads were observed and, thus, a better fragment distribution and a higher number of transcripts were analysed; 2) the transcriptome complexity has been reported [35] to be higher in kidney, testes and brain tissues in comparison to liver and muscle.

### Gene orthology and lncRNAs detection

From the unannotate (Sscrofa 9.61 assembly) intergenic expressed regions, a range of 3488–5658 intergenic novel transcripts were identified in each sample (Table 4). However, to find not annotated genes, transcripts expressed in at least four of the five animals of each group were considered. Then, Augustus software [36] was used to examine which of these transcripts were predicted to encode proteins. A total of 146 and 180 putative proteins were identified for the L and H groups, respectively (Table 5). According to BLASTP results, these proteins correspond for L group to: 19 novel computationally predicted and 95 known human proteins, 3 novel and 107 known bovine proteins and 43 novel and 7 know-porcine proteins. Similarly, for the H group predicted proteins correspond to: 25 novel computationally predicted and 110 known human proteins, 5 novel and 128 known bovine proteins and 51 novel and 8 known porcine proteins. Interestingly, in both H and L groups, around 86% of the predicted novel proteins were found in the *Sus scrofa* protein database ftp://ftp.ensembl.org/pub/release-65/fasta/sus_scrofa/pep/ as novel computationally predicted proteins. Moreover, the number of matches increased to 58% (104/180) when the predicted novel proteins were compared against the putative coding transcripts reported by Esteve-Codina *et al*., (2011). This result indicates that a high number of genes are not annotated in the Sscrofa 9.61 pig genome assembly and they are expressed in both liver and gonad tissues. Finally, these results constitute an experimental confirmation of the novel computationally predicted genes in pigs.

**Table 5:** **Putative proteins identified in each H and L groups and orthologies detected against* Homo sapiens*,* Bos taurus* and* Sus scrofa* protein databases**

**Group**	**Total**	***Bos taurus***	***Homo sapiens***	***Sus scrofa***	**Esteve-Codina et al. (2011)**
*L*	146	110	114	50	82
*H*	180	133	135	59	104

Predicted proteins reported in pigs by Esteve-Codina et al., (2011) are also included.

For lncRNAs annotation, the previously reported sequences in pig male gonad transcriptome [23] were used as a reference database. A total of 186 (L) and 270 (H) of these putative lncRNAs was also expressed in pig liver. Within groups, 101 and 108 lncRNAs were expressed in all L and H animals, respectively, but only 89 lncRNAs were expressed in both groups (Additional file 5, Figure S3).

### Differential gene expression analysis

**Figure 2:**
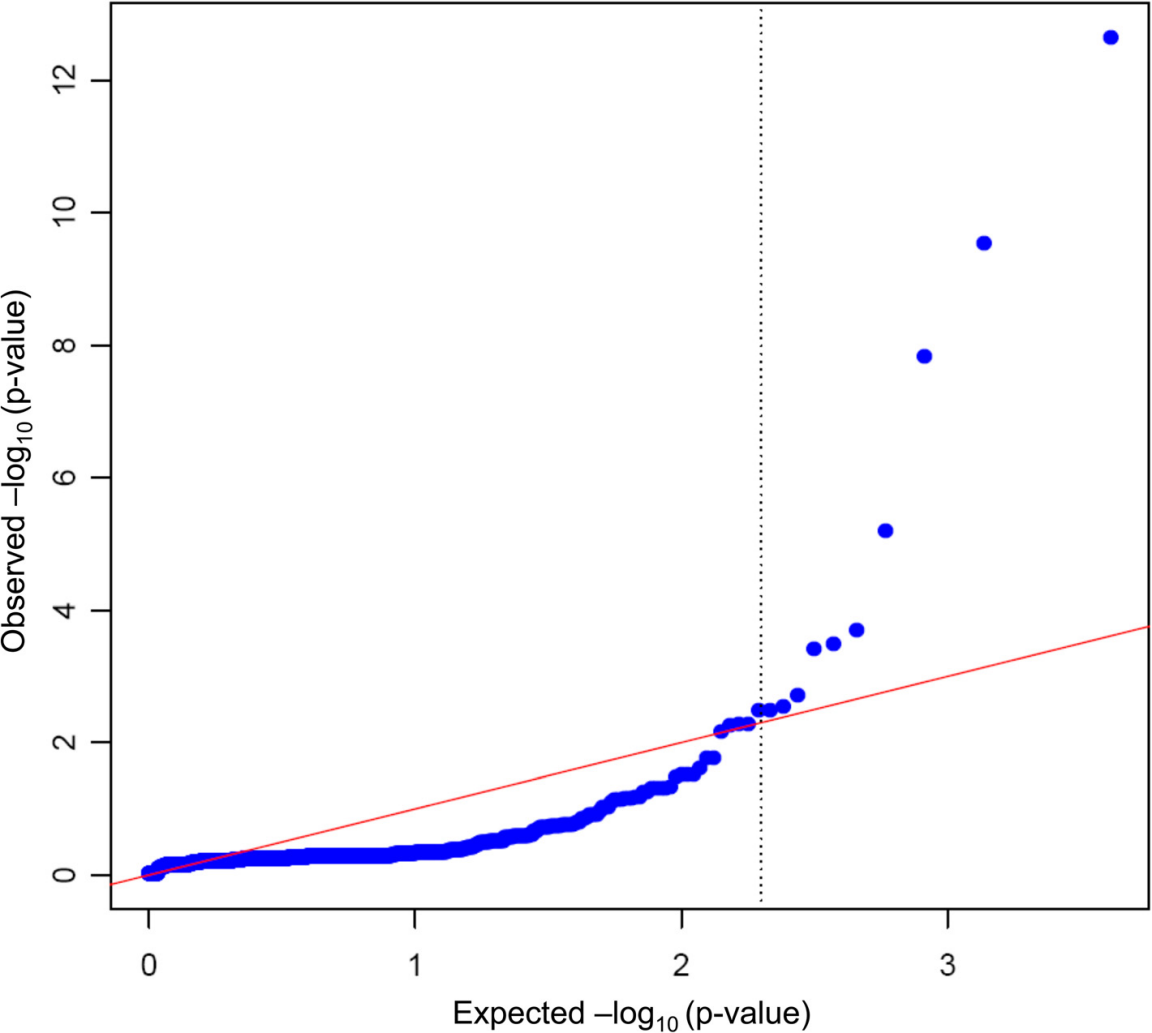
**Q-Q plot representing the distribution of the p-value.** Red line represents the expected distribution of the p-value, while blue trend represents the observed distribution. X-axis values are Expected -log_10_ (p-value) and y-axis values are the Observed –log _10_ (p-value).

**Figure 3:**
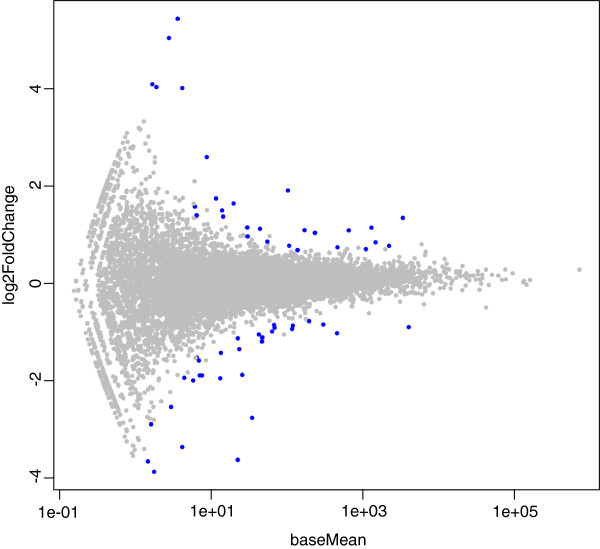
**Plot of the 55 differentially-expressed protein-coding genes (represented in blue) with fold change ≥ 1.5 and p-value ≤ 0.005.** X-axis values are base mean expression values and y-axis values are the log2 (fold change).

DESeq software [37] was employed to detect differentially-expressed (DE) genes between H and L groups. First, some exploratory analyses to estimate the variance and quality of the data were performed. Per-gene estimates of the base variance against the base levels showed that the fit (red line) closely followed the single-gene estimates (Additional file 6, Figure S4). The 'residualsEcdfPlot' function, which checks the uniformity of the cumulative probabilities, revealed a similar pattern for the curves of the empirical cumulative density functions (ECDF) in both groups (Additional file 7, Figure S5). It was also noted that ECDF followed well the diagonal, except for very low counts, but that is to be expected because at this level shot noise dominates and, therefore, the deviations become stronger (Additional file 8, Figure S6). Afterwards DE analysis between groups was performed. Figure 2 shows that, at selected cut-off (−log10(p-value)>2.3 or q-value ≤ 0.17), there is a clear departure from expected among transcripts accepted as differentially expressed (indicated by the blue trend being above the strait red line). Therefore according to the employed cut-off (fold change ≥ 1.5 and p-value ≤ 0.005), 55 protein-coding genes, two pseudogenes [ENSEMBL_Id: ENSSSCG00000004170, ENSSSCG00000016238] and one non-coding RNA [ENSEMBL_Id: ENSSSCG00000019010] were identified as differentially-expressed (Figure 3, Table 6).

**Table 6:** Description of the differentially-expressed genes detected between High and Low groups with fold change ≥ 1.5 and p-value ≤0.005

**Ensembl_Gene_Id**	**Human homolog**	**Fold change**	**p-value**	**q-value**	**Gene biotype**
ENSSSCG00000010610	GSTO1	3.8	1.1 x 10^-16^	2.2x10^-13^	protein_coding
ENSSSCG00000010992	AQP7	−6.7	3.0 x 10^-13^	2.9 x 10^-10^	protein_coding
ENSSSCG00000016401	KIF1A	−12.5	2.2 x 10^-11^	1.4 x 10^-8^	protein_coding
ENSSSCG00000010488	CYP2C9	2.5	1.3 x 10^-8^	6.3 x 10^-6^	protein_coding
ENSSSCG00000013865	NWD1	16.2	5.2 x 10^-7^	2.0 x 10^-4^	protein_coding
ENSSSCG00000012015	C21orf91	20.4	1.0 x 10^-6^	3.0 x 10^-4^	protein_coding
ENSSSCG00000007873	--	−3.7	1.4 x 10^-6^	4.0 x 10^-4^	protein_coding
ENSSSCG00000009871	SDS	2.2	7.8 x 10^-6^	2.0 x 10^-3^	protein_coding
ENSSSCG00000002383	FOS	−2.0	1.3 x 10^-5^	3.0 x 10^-3^	protein_coding
ENSSSCG00000019010	--	−14.3	1.6 x 10^-5^	3.0 x 10^-3^	snRNA
ENSSSCG00000000044	C22orf32	−3.8	1.8 x 10^-5^	3.0 x 10^-3^	protein_coding
ENSSSCG00000003891	CYP4A11	2.1	3.2 x 10^-5^	1.0 x 10^-2^	protein_coding
ENSSSCG00000016238	--	3.1	3.5 x 10^-5^	1.0 x 10^-2^	pseudogene
ENSSSCG00000011937	MORC1	6.1	3.9 x 10^-5^	1.0 x 10^-2^	protein_coding
ENSSSCG00000010487	CYP2C19	2.1	5.1 x 10^-5^	1.0 x 10^-2^	protein_coding
ENSSSCG00000006614	THEM5	18.0	1.4 x 10^-4^	2.0 x 10^-2^	protein_coding
ENSSSCG00000005385	NR4A3	−2.7	1.4 x 10^-4^	2.0 x 10^-2^	protein_coding
ENSSSCG00000006580	S100A2	−3.7	2.2 x 10^-4^	2.0 x 10^-2^	protein_coding
ENSSSCG00000000231	ANKRD33	−2.6	3.0 x 10^-4^	3.0 x 10^-2^	protein_coding
ENSSSCG00000001642	TBCC	−14.3	3.2 x 10^-4^	3.0 x 10^-2^	protein_coding
ENSSSCG00000003971	--	−2.0	3.3 x 10^-4^	3.0 x 10^-2^	protein_coding
ENSSSCG00000015294	CR1	−2.0	3.7 x 10^-4^	3.0 x 10^-2^	protein_coding
ENSSSCG00000006238	CYP7A1	2.1	5.6 x 10^-4^	5.0 x 10^-2^	protein_coding
ENSSSCG00000004789	THBS1	−1.7	6.0 x 10^-4^	5.0 x 10^-2^	protein_coding
ENSSSCG00000012832	MXRA5	−2.1	6.5 x 10^-4^	5.0 x 10^-2^	protein_coding
ENSSSCG00000004946	ZWILCH	2.2	6.7 x 10^-4^	5.0 x 10^-2^	protein_coding
ENSSSCG00000007888	TNFRSF17	−3.7	6.7 x 10^-4^	5.0 x 10^-2^	protein_coding
ENSSSCG00000008595	APOB	1.6	7.9 x 10^-4^	5.0 x 10^-2^	protein_coding
ENSSSCG00000014919	ME3	−2.3	8.4 x 10^-4^	6.0 x 10^-2^	protein_coding
ENSSSCG00000014368	--	−2.0	1.0 x 10^-3^	7.0 x 10^-2^	protein_coding
ENSSSCG00000007529	SYCP2L	2.6	1.0 x 10^-3^	7.0 x 10^-2^	protein_coding
ENSSSCG00000013116	--	16.4	1.1 x 10^-3^	7.0 x 10^-2^	protein_coding
ENSSSCG00000004052	FNDC1	−1.8	1.2 x 10^-3^	7.0 x 10^-2^	protein_coding
ENSSSCG00000002277	SPTB	−4.0	1.2 x 10^-3^	7.0 x 10^-2^	protein_coding
ENSSSCG00000016645	C7orf53	−7.7	1.3 x 10^-3^	7.0 x 10^-2^	protein_coding
ENSSSCG00000008203	IGKV2-40	−1.8	1.3 x 10^-3^	7.0 x 10^-2^	protein_coding
ENSSSCG00000001229	--	−2.0	1.3 x 10^-3^	7.0 x 10^-2^	protein_coding
ENSSSCG00000000151	APOL6	3.4	1.5 x 10^-3^	8.0 x 10^-2^	protein_coding
ENSSSCG00000004170	--	2.2	1.9 x 10^-3^	8.0 x 10^-2^	pseudogene
ENSSSCG00000016190	SLC11A1	−3.8	1.9 x 10^-3^	8.0 x 10^-2^	protein_coding
ENSSSCG00000006355	APOA2	1.8	1.9 x 10^-3^	8.0 x 10^-2^	protein_coding
ENSSSCG00000015747	MYOM2	2.8	2.3 x 10^-3^	9.0 x 10^-2^	protein_coding
ENSSSCG00000014824	RELT	−12.5	2.7 x 10^-3^	9.0 x 10^-2^	protein_coding
ENSSSCG00000008455	ABCG8	17.1	2.7 x 10^-3^	9.0 x 10^-2^	protein_coding
ENSSSCG00000003777	SLC44A5	2.6	2.8 x 10^-3^	9.0 x 10^-2^	protein_coding
ENSSSCG00000003999	A1BG	−5.9	2.8 x 10^-3^	9.0 x 10^-2^	protein_coding
ENSSSCG00000002375	RPS6KL1	−2.2	3.2 x 10^-3^	1.3 x 10^-1^	protein_coding
ENSSSCG00000001006	TUBB2B	−2.9	3.4 x 10^-3^	1.4 x 10^-1^	protein_coding
ENSSSCG00000007478	ATP9A	−1.8	3.5 x 10^-3^	1.4 x 10^-1^	protein_coding
ENSSSCG00000009992	UQCR10	1.8	3.9 x 10^-3^	1.6 x 10^-1^	protein_coding
ENSSSCG00000010829	MOSC1	1.7	4.0 x 10^-3^	1.6 x 10^-1^	protein_coding
ENSSSCG00000017923	ALOX15	−3.0	4.3 x 10^-3^	1.6 x 10^-1^	protein_coding
ENSSSCG00000002847	GPT2	1.7	4.6 x 10^-3^	1.7 x 10^-1^	protein_coding
ENSSSCG00000004787	GPR176	3.0	4.7 x 10^-3^	1.7 x 10^-1^	protein_coding
ENSSSCG00000006985	MTMR7	1.7	4.8 x 10^-3^	1.7 x 10^-1^	protein_coding
ENSSSCG00000000709	PLEKHG6	−1.9	5.0 x 10^-3^	1.7 x 10^-1^	protein_coding
ENSSSCG00000010892	KCNT2	2.0	5.0 x 10^-3^	1.7 x 10^-1^	protein_coding
ENSSSCG00000008624	LPIN1	1.6	5.0 x 10^-3^	1.7 x 10^-1^	protein_coding

In order to validate the expression data obtained by RNA-Seq, five genes (*APOA2*, *LPIN1*, *ME3*, *CYP7A1* and *CYP2C49*) were selected among the differentially-expressed protein-coding genes to perform real time reverse transcription (RT-qPCR) assays. When the pattern of gene expression levels was compared, strong correlations ranking from 0.79 to 0.96 between RT-qPCR and RNA-Seq platforms were observed, confirming the high reproducibility of the data (Additional file 9, Table S3).

Interestingly, one of the studied genes, the *CYP2C49* [ENSEMBL_Id: ENSSSCG00000010488] which belongs to the highly diverse superfamily of CYP450 [38] and it is homologue to the human *CYP2C9* gene, was located in a genomic region in which copy number variation (CNV) has been previously described in pigs [39]. In order to assess whether observed differences of gene expression were influenced by differences in the CNV between animals, a real time quantitative PCR (qPCR) to determine the number of copies of the *CYP2C49* gene was developed. For the first time, CNV affecting the *CYP2C49* gene was described with relative quantification values ranging from 1 to 5.2 copies (Additional file 9, Table S3). However, no correlation between the number of copies and gene expression was observed. Therefore, further analysis will be necessary to elucidate the possible role of these structural variants in the fatty acid metabolism.

Moreover, it is noteworthy that six of the differentially-expressed genes related to fatty acids metabolism in liver (*APOB, CYP7A1, APOA2, THBS1, THEM5, ME3)* were previously reported as associated with the profile of intramuscular fatty acid composition in a GWAS study in the same animal population [30]. Therefore, they can be considered as interesting candidate genes and this suggest their role in the fatty acid metabolism processes in pigs in both liver and IMF tissues (Table 7).

**Table 7:** Differentially-expressed genes previously reported to be associated with the profile of intramuscular fatty acid composition in a genome-wide association study

**Ensembl gene ID**	**Chr**	**Start (bp)**^**1**^	**End (bp)**^**1**^	**Gene name**	**Fatty acid**
ENSSSCG00000004789	1	138129409	138145238	THBS1	C18:1(n-9), C18:2(n-6), MUFA
ENSSSCG00000008595	3	109052838	109076900	APOB	C16:1(n-7), ratio C16:1(n-7)/c16:0
ENSSSCG00000006238	4	77173363	77202771	CYP7A1	C16:1(n-7), C18:2(n-6)
ENSSSCG00000006355	4	92745976	92747590	APOA2	C16:0, C18:2(n-6), rate MUFA/SFA
ENSSSCG00000006614	4	101212520	101222091	THEM5	rate MUFA/SFA
ENSSSCG00000014919	9	20546397	20647476	ME3	rate C20:1/C20:0

^1^ The genomic coordinates are expressed in bp and are relative to the *Sus scrofa* April 2009 genome sequence assembly (Sscrofa9).

### Functional clustering of differentially-expressed genes in the liver

From the 55 differentially-expressed protein-coding genes, 26 were up-regulated and 29 were down-regulated in H group in comparison to L (Table 6). To gain insight into the liver tissue processes that differed between groups, the list of the differentially-expressed genes was explored using the core analysis function included in Ingenuity Pathways Analysis (IPA). Initially, the pig gene IDs were converted to human genes but five protein-coding genes did not match with human homologs [Ensembl Ids: ENSSSCG00000007873, ENSSSCG00000003971, ENSSSCG00000014368, ENSSSCG00000013116, ENSSSCG00000001229], and therefore only 50 pig genes were eligible for network construction.

The top seven biological functions identified by IPA included categories related to a wide variety of physiological and biological events, such as lipid metabolism, small molecule biochemistry, molecular transport, drug metabolism, energy production, nucleic acid metabolism, and vitamin and mineral metabolism (Table 8). A specific examination of the lipid metabolism IPA molecular and cellular function revealed that most of the transcripts relating to lipid metabolism were up-regulated in H group compared to L group. Remarkably, genes that play a crucial role in lipoprotein synthesis *(APOB*), cholesterol metabolism (*ABCG8, CYP2C9, CYP2C19, CYP4A11, APO* and *CYP7A1*), oxidation of lipids and palmitic fatty acids (*MTMR7*), and induction of lipogenic gene transcription (*LPIN1*) were up-regulated in H group in contrast to L group. On the contrary, genes involved with accumulation of triacylglycerol (*AQP7*), uptake of lipids and myristic acid (*THBS1*), and fatty acids biosynthesis (*ME3*) were down-regulated in H group as opposed to L group. The malic enzyme is involved in supplying NADPH for the reductive biosynthesis of fatty acids [40]. Based on the above observations it is tempting to speculate that ALA and LA acids reaching liver tissue inhibit the expression of *ME3* gene, and consequently, at least partially, reduce lipogenic activity. This is in agreement with Guillevic et al. [8], who reported that ALA acid enriched diets decreased malic enzyme activity in liver and subcutaneous adipose tissue of pigs.

**Table 8:** Description of the top seven molecular and cellular biological functions significantly modulated in the liver tissue when comparing H relative to L animals

**Category**	**Genes**	**p-value**
Lipid Metabolism	*ABCG8,ALOX15,AQP7,APOB,CYP2C9,THBS1, APOA2,ME3,NR4A3,LPIN1,CYP7A1,MTMR7, CYP2C19,CYP4A11*	1.15x10 ^-7^
Small Molecule Biochemistry	*ABCG8,ALOX15,AQP7,APOB,CYP2C9,THBS1, APOA2,ME3,GSTO1,FOS,NR4A3,LPIN1,SPTB, CYP7A1,MTMR7,CYP2C19,SDS,SLC11A1, CYP4A11*	1.15x10 ^-7^
Molecular Transport	*ABCG8,AQP7,ALOX15,APOB,THBS1,APOA2, GSTO1,FOS,NR4A3,LPIN1,CYP7A1,SLC11A1, FNDC1*	2.62x10 ^-6^
Drug Metabolism	*FOS,CYP2C9,THBS1,CYP2C19*	7.20x10 ^-6^
Energy Production	*NR4A3,LPIN1,CYP2C9,APOA2,ME3, CYP2C19,SDS,CYP4A11*	7.20x10 ^-6^
Nucleic Acid Metabolism	*CYP2C9,THBS1,CYP2C19*	5.85x10 ^-6^
Vitamin and Mineral Metabolism	*ABCG8,APOB,CYP2C9,APOA2,CYP7A1, CYP2C19,GSTO1*	8.65x10 ^-6^

Statistical significance of pathway modulation was calculated via a right-tailed Fisher’s Exact test in Ingenuity Pathway and represented as –log (*P* value): -log values exceeding 1.30 were significant false discovery rate (FDR) < 0.05.

**Figure 4:**
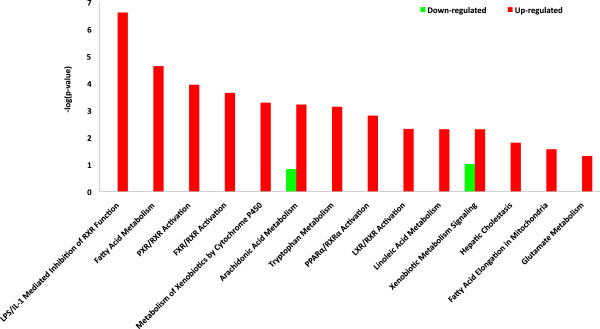
**Canonical pathway significantly detected when contrasting the up (red) and (green) down-regulated genes in H compared to L group.** X-axis values are the log(B-H correction p-value) and Y-axis values are the canonical pathways. The statistical significance of pathway modulation was calculated via a right-tailed Fisher’s Exact test in Ingenuity Pathway analysis and represented as –log (*P* value): -log values exceeding 1.30 were significant false discovery rate (FDR) <0.05).

Interestingly, the most representative canonical pathways significantly modulated in liver when comparing H vs L groups were involved in Endotoxin lipopolysaccharide / pro-inflammatory cytokines (LPS/IL-1) mediated inhibition of retinoid X receptors (RXR) function, fatty acid metabolism (including AA and LA acids) and pregnane X receptor / farnesoid X receptor (FXR/RXR) activation (Figure 4 and Table 9), in which the up-regulation of *ABCG8, APOB and CYP7A1* genes was observed. Likewise, the present findings underscore that H group increased the expression of gene sets regulated by peroxisome proliferator-activated receptors alpha (PPAR-α) *(APOA2, CYP2C9, CYP2C19)* and RXR (*ABCG8, CYP7A1*) transcription factors, both of which are shown to have an important role in lipid homeostasis. For instance, PPAR-α is an important regulator of cellular fatty acid uptake and intracellular fatty acid transport, mitochondrial and peroxisomal fatty acid oxidation, ketogenesis, and gluconeogenesis in several species [41-43], whereas *RXR* plays a crucial role in the transcriptional regulation of a spectrum of genes controlling cholesterol homeostasis and bile acid homeostasis, together with nuclear receptor FXR, a key transcription factor that regulates cholesterol 7 α-hydroxylase (*CYP7A1*) activity and mRNA levels.

**Table 9:** Description of the top six canonical pathways significantly modulated in liver tissue when comparing H to L animals

**Ingenuity canonical pathways**	**Genes**
LPS/IL-1 Mediated Inhibition of RXR Function	*ABCG8,CYP2C9,CYP7A1,CYP2C19, CYP4A11,GSTO1*
Arachidonic Acid Metabolism	*ALOX15,CYP2C9,CYP2C19,CYP4A11*
Fatty Acid Metabolism	*CYP2C9,CYP2C19,SDS,CYP4A11*
PXR/RXR Activation	*CYP2C9,CYP7A1,CYP2C19*
Linoleic Acid Metabolism	*ALOX15,CYP2C9,CYP2C19*
FXR/RXR Activation	*ABCG8,APOB,CYP7A1*

Statistical significance of pathway modulation was calculated via a right-tailed Fisher’s Exact test in Ingenuity Pathway analysis and represented as –log (*P* value): -log values exceeding 1.30 were significant FDR <0.05.

In addition, the up-regulation of *PPAR*-α and *RXR* were coupled with the increased expression of *lipin* (*LPIN1*) and *CYP7A1* genes. In mice, it has been reported that *LPIN1* selectively activates a subset of coactivator 1α (*PGC-1α*) target pathways involved in fatty acid oxidation and mitochondrial oxidative phosphorylation, while suppressing the lipogenic program and lowering circulating lipid levels [44]. Lipin activates mitochondrial fatty acid oxidative metabolism by inducing expression of the nuclear receptor *PPAR-α*, a known *PGC-1α* target, and via direct physical interactions with *PPAR-α* and *PGC-1α*. Furthermore, *CYP7A1* has been shown to be a key factor of hepatic cholesterol homeostasis. All together these results suggest that H group may present greater uptake of fatty acids into hepatocytes (mainly LA and ALA acids). It is likely that the higher PUFA bioavailability in liver may affect expression of PPAR-α, RXR and their target genes, inducing a greater stimulation of both peroxisomal and mitochondrial β-oxidation, and leading to reduced triglyceride and cholesterol synthesis, and an enhanced elimination of cholesterol from the liver via bile acid formation. This intriguing possibility remains to be demonstrated, although there is evidence that FA, in particular unsaturated FA, exert many of their biological effects by regulating the activity of numerous transcription factors in liver, such as PPAR-α [45]. Recently, [46] has demonstrated that FA oxidation is regulated by hepatic MUFA to PUFA ratio through the activation of *PPAR-α.* In agreement to our results, hepatic expression of *PPAR-α* was higher in pigs fed with a higher level of PUFA. This is also in line with the lower IMF content in H group than in L animals, and the lower proportion of *de novo* fatty acids in the IMF. Therefore, these transcriptome changes may reflect counter mechanisms of liver tissue to respond or compensate for changes in IMF fatty acid profile, which depends on possible metabolisation of FAs and the possibility of being synthesized by the pig adipose tissue [47]. However, the question remains how different types of FA control the expression of genes and a direct examination of the effect of each individual FA on porcine muscle fatty acid composition is needed.

**Figure 5:**
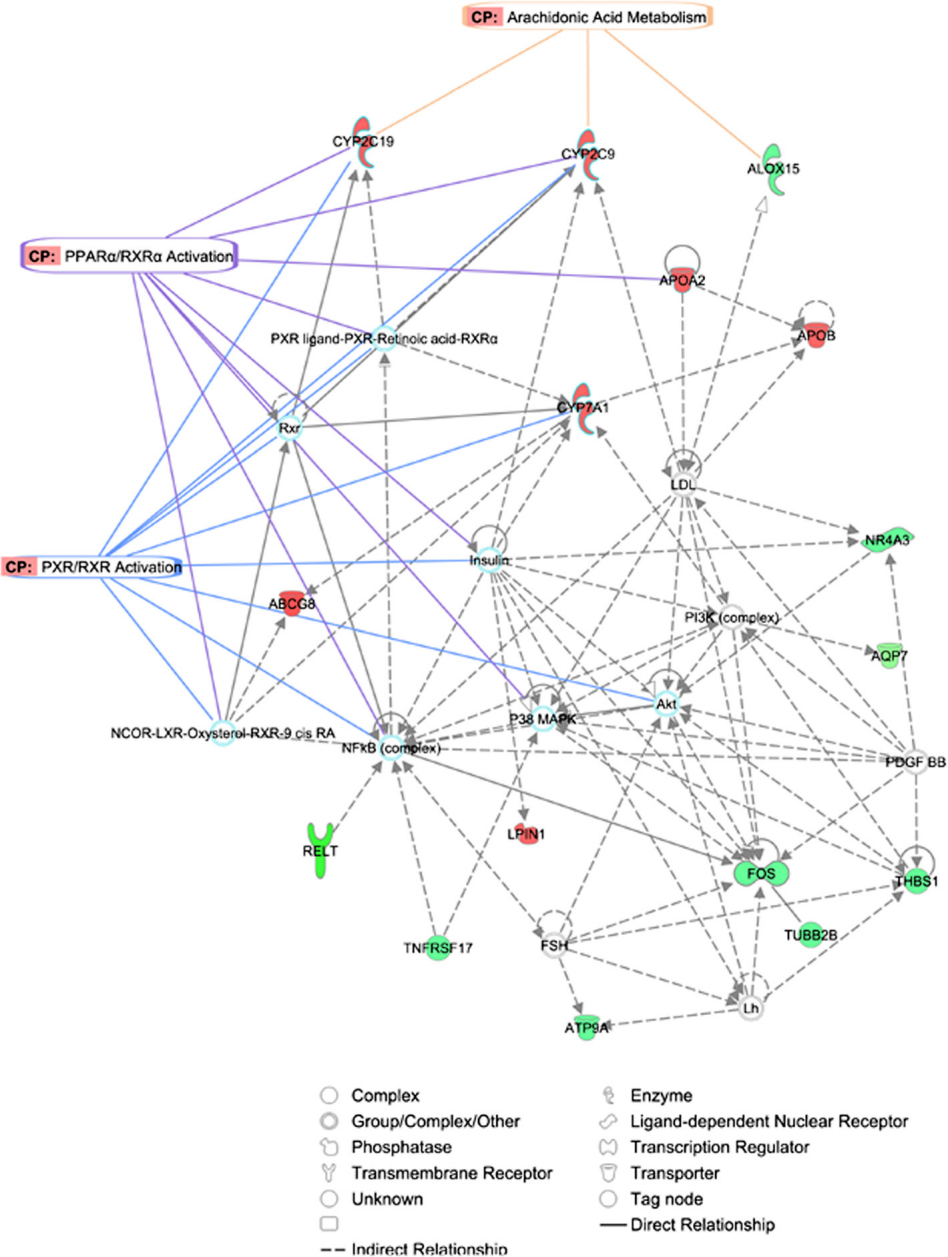
**Network 1 as generated by IPA.** The significant biological functions comprising this network are lipid metabolism, small molecule biochemistry and vitamin and mineral metabolism. The network is displayed graphically as nodes (gene/gene products) and edges (the biological relationship between nodes). The node colour indicates the expression of genes: (red) up-regulated and (green) down-regulated in H group relative to L group. The shapes of nodes indicate the functional classes of the gene products. Relevant canonical pathways that feature modulated genes were indicted as well (e.g. Arachidonic Acid metabolism, PPARα/RXRα and PXR/RXR Activation).

**Figure 6:**
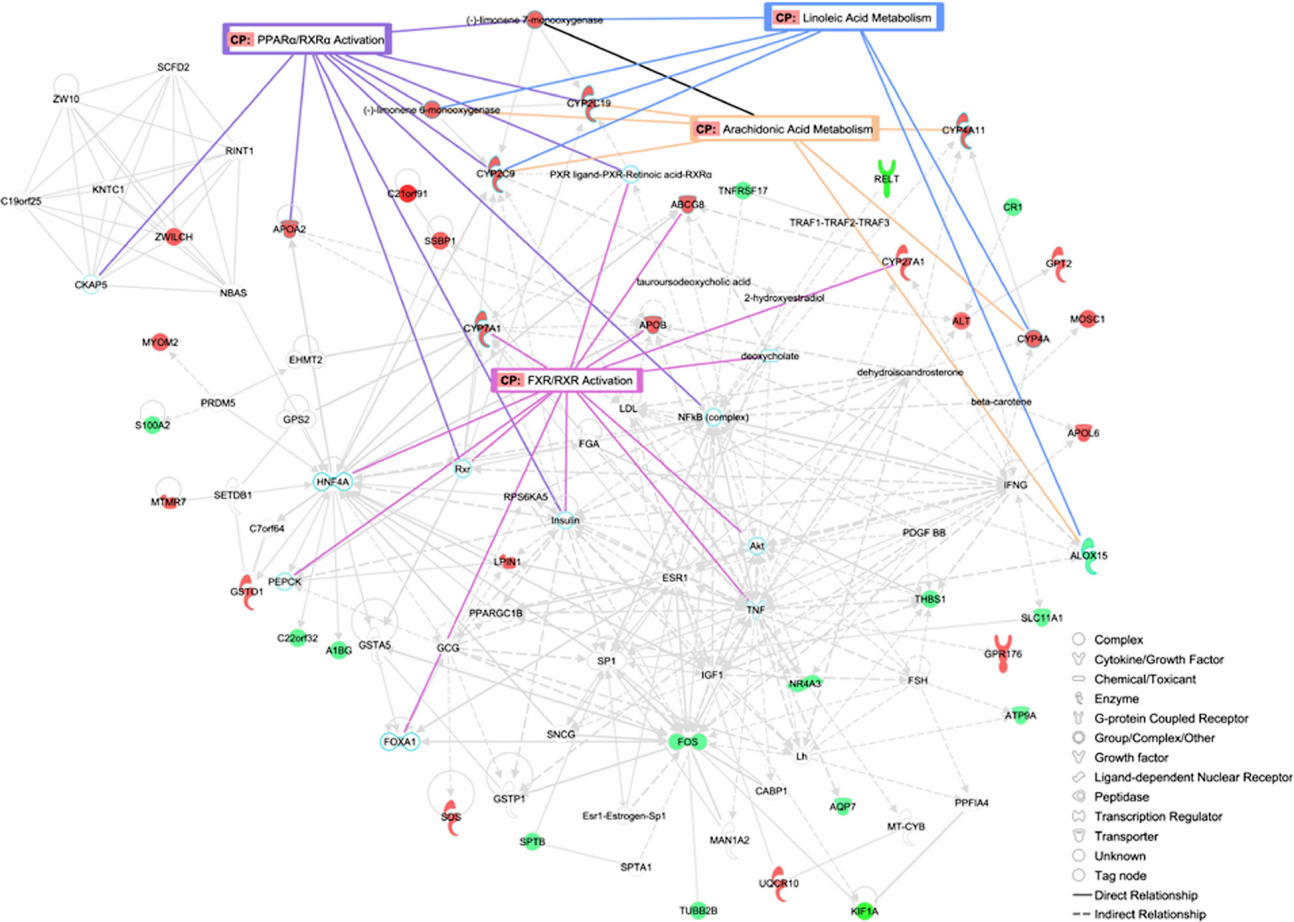
**Graphical representation of the three merged networks generated by IPA.** Depicted is the result of merging the network 1 (lipid Metabolism, Small Molecule Biochemistry and Vitamin and Mineral Metabolism), network 2 (lipid Metabolism, Molecular Transport and Small Molecule Biochemistry) and network 3 (carbohydrate Metabolism, Lipid Metabolism and Molecular Transport). The overrepresented canonical pathways such as Arachidonic and Linoleic Acid metabolism, PPARα/RXRα and FXR/RXR Activation are overlaid onto the resulting network, to show which genes are directly involved in these significant processes. Genes and gene products are represented as nodes and the relationship among these is represented as a line. Red indicates Up-regulated and green Down-regulated expression of genes when comparing H to L groups.

Finally, the identified genes were mapped to three genetic networks. The first, having an IPA network score of 38 and 16 focus genes, presented functions related to Lipid Metabolism, Small Molecule Biochemistry and Vitamin and Mineral Metabolism (Figure 5). The second, with a score of 23 and 11 focus genes centred on Lipid Metabolism, Molecular Transport and Small Molecule Biochemistry (Additional file 10, Figure S7), and the third network scoring 21 and 10 focus genes was associated with Carbohydrate Metabolism, Lipid Metabolism and Molecular Transport (Additional file 11, Figure S8). When the top three IPA networks were merged a connection between them was observed (Figure 6), suggesting that the differentially-expressed genes detected in this study are linked and play an important role in lipid metabolism. Remarkably, IPA results are in conformity with the design of this experiment, which inferred that LA and AA acids metabolism were altered between the groups of sequenced individuals.

### Implications

In the present study, RNA-Seq was used for the analysis of the pig liver transcriptome in animals of extreme phenotypes for intramuscular fatty acid composition.

The liver plays an important role in lipid metabolism and, thus, the analysis of liver transcriptome in extreme pigs for intramuscular fatty acid composition may be relevant to elucidate its functional complexity. Although the main goal of this study was to find differentially-expressed genes between phenotypically extreme animals, the use of RNA-Seq allowed the identification of transposable elements, lncRNAs and new protein-coding genes in the porcine liver transcriptome.

The first principal component of PCA analysis classified animals in two extreme groups for the fatty acid composition of LD muscle. Group H of animals had a higher content of PUFA, including essential FA such as LA, ALA, ELE and AA acids than group L animals. Conversely, the latter had a higher content of SFA and MUFA, palmitoleic and oleic acids.

The lipid content and fatty acid profile of muscle plays an important role in the tenderness, flavour and juiciness of cooked meat [6]. In swine production, the reduction of intramuscular fat (IMF) in some breeds due to a preference selection for lean pigs, has affected meat quality. From this point of view, PUFA has a negative effect on the oxidative stability of muscle, which, in turn, affects flavour and muscle colour [6]. On the other hand, desirable sensorial characteristics tend to be associated with MUFA and SFA [6,48,49]. Lipid and fatty acid compositions of food have an important impact on human health, with a high consumption of SFA associated with obesity, high plasma cholesterol and cardiovascular disease [50,51]. Conversely, PUFAs, mainly ω−3, have been considered beneficial for human health, by reducing serum low-density lipoprotein-C, total cholesterol concentration and modulating immune functions and inflammatory processes [52-54].

There is increasing awareness of the wide range of health benefits of PUFA in general, and of ω−3 fatty acids in particular. Meat is an important basis of human nutrition, and pork meat is seen to be a major source of human food. The composition of fatty acids stored in adipose tissue in pigs largely reflects that of ingested lipids [5]. Thus, swine meat enriched with ω−3 fatty acids can be achieved by feeding with commercial diets supplemented with this PUFA [8], and possibly by selective breeding. In fact, there is a genetic basis of PUFA level in pork meat. It is likely that H group presented higher absorption of essential PUFA, increasing their amount reaching the IMF tissue, which in turn could be considered as an important factor in the inhibition of the *de novo* saturated fatty acid proportion in meat. Furthermore, differences on elongation, desaturation and oxidation of those essential PUFA to longer-chain ω−3 and ω−6 fatty acids cannot be discarded. Therefore, from the human health perspective, increasing H genotypes through breeding programs could be desirable because meat and meat-derived foods are still large contributors to saturated fatty acids intake in humans. These observations, together with several gene expression effects are the major factors leading us to believe that genetic was indeed a significant factor affecting meat IMF PUFA content and composition. However, an inverse relationship exists between nutritional value and eating quality of meat and, as consequence, established selection criteria to all together improve meat quality from the sensorial and nutritional point of view is a complex matter. Therefore, a holistic approach including both nutrigenetic and nutrigenomic disciplines may be required to improve the pork meat quality from both points of view.

## Conclusions

We used RNA-Seq as a tool to explore the liver transcriptome of ten female pigs with extreme phenotypes for intramuscular fatty acid composition. Transposable elements, lncRNAs and new putative protein-coding genes were identified. Reproducibility of the data was confirmed by the strong correlation observed between the values of gene expression obtained by RNA-Seq, RT-qPCR and microarrays. A total of 55 genes differentially-expressed between extreme animals were identified. These genes belong to canonical pathways and gene networks related to the lipid and fatty acid metabolism. In concordance with the initial phenotypic classification, pathway analysis inferred that linolenic and arachidonic acid metabolism was altered between extreme animals. The results obtained may help in the design of new selection strategies to improve pork meat quality from both the sensorial and nutritional points of view.

## Methods

### Animal material and phenotypes

The population studied was originated by crossing three Iberian boars (Guadyerbas line) with 31 Landrace sows [55,56]. Five F1 males were backcrossed with 26 Landrace sows and 144 BC1_LD pigs were obtained. All pigs were raised in a normal intensive system, fed under standard management conditions and were slaughtered at an average age of 179.8 ± 2.6 days following national and institutional guidelines for the ethical use and treatment of animals in experiments.

A total of 48 traits related with growth, carcass quality and intramuscular fatty acid composition were measured. A PCA was performed with the *prcomp* procedure of R software [57], including phenotypic information from twenty-six of the total traits. Four of these were related to carcass quality (carcass height, weight of ham, weight of shoulder and intramuscular fat) whereas the rest corresponded to fatty acids composition in muscle and indices of fatty acids metabolism. Animals with extreme phenotypes, according to the first principal component, were selected to generate the High (H) and Low (L) groups with 20 animals per group (Figure 1). Phenotypic mean comparisons between groups were performed using R. Since sex differences in liver transcriptome have been reported in several species[58], selection was made considering pedigree information representing the parental genetic diversity and only females were retained for RNA sequencing (five per group).

### RNA isolation, library preparation and sequencing

From the 10 selected animals, total RNA was isolated from liver using the *RiboPureTM Isolation of High Quality Total RNA* (*Ambion®, Austin, TX*) following the manufacturer’s recommendations. RNA was quantified using the *NanoDrop ND-1000 spectrophotometer* (*NanoDrop products*, Wilmington, USA) and checked for purity and integrity in a *Bioanalyzer-2100* (*Agilent Technologies, Inc*., Santa Clara CA, USA).

Sequencing libraries were generated using *Illumina mRNA-Seq* following manufacturer’s instructions and ten index codes were added to attribute sequences to each animal. A total of two channels of *an Illumina Hi-Seq 2000* instrument (*Fasteris SA*, Plan-les-Ouates, Switzerland) were used to sequence two pools of five samples (one pool with five samples per lane with barcoding).

### Mapping, assembling and annotation of reads

After removal of sequencing adaptors and low-complexity readsTopHatv1.2.0 software [31] was employed to map reads using as reference the version 9.61 of pig genome (Sscrofa 9.61) http://www.ensembl.org/info/data/ftp/index.html. Quality control and reads statistics were determined with FASTQC http://www.bioinformatics.bbsrc.ac.uk/projects/fastqc/. Transcript assembly was performed using Cufflinks v0.9.3 [59] , with a minimum alignment count per *locus* of 10. Finally, S-MART http://urgi.versailles.inra.fr/Tools/S-MART for read annotation was used.

### Gene expression quantification and correlation analysis with expression microarrays

Gene expression quantification was performed using the normalized number of fragments per kilobase of exon per million reads (FPKM) as reported in Cufflinks output [59]. Correlations between mean expression values between groups were calculated. All individuals were also assayed with high-density oligonucleotide microarray chips (*GeneChip® Porcine*) from *Affymetrix* (Santa Clara, CA) containing a total of 23,937 probe sets (23,256 transcripts), representing 20,201 *Sus scrofa* genes. Microrarrays were hybridized and scanned at the *Institut de Recerca Hospital Universitari Vall d’Hebron* (Barcelona, Spain) following *Affymetrix* standard protocols. Expression data were generated with Gene-Chip Operating Software (GCOS). Probes were adjusted for background noises and normalized using the GCRMA R package [60]. The average probe value per gene was calculated and a total of 6,025 Ensembl gene IDs could be retrieved to estimate the Spearman correlation between the log2 expression values of genes analysed by RNA-Seq and microarrays. Finally, a GO enrichment analysis with the QuickGO browser http://www.ebi.ac.uk/QuickGO/ was performed for the top 100 most expressed genes.

### Differential gene expression analysis

Differential expression analysis (DE) between groups was performed using DESeq [37]. This R package uses as input file the unambiguous table of counts per gene obtained from HTseq-count http://www-huber.embl.de/users/anders/HTSeq/doc/overview.html. DESeq models the data using negative binomial distributions assuming that the mean is a good predictor of variance. Therefore, it assumes that genes with similar expression level also have similar variance across replicates [37]. Following the DESeq author’s recommendations, some exploratory diagnostic plots were executed to check the dispersion estimate and data quality. In order to ascertain the base variance the function 'varianceFitDiagnostics' was used and the per-gene estimates of the base variance was plotted against the base levels. The uniformity of the cumulative probabilities estimated by the 'varianceFitDiagnostics' was also verified via the 'residualsEcdfPlot' function.

Differentially-expressed genes were detected through the ‘nbinomTest’ function of DESeq. All the genes with a fold change between H and L groups higher than or equal to 1.5 fold were retained (total of 2051 genes). Then, for this subset of genes, the R package q-value [61] was employed to calculate the false-discovery rate and genes with a p-value ≤ 0.005 (which is equivalent to a q-value ≤ 0.17) were retained.

### Validation of differentially-expressed genes by RT-qPCR and copy number determination by qPCR

In order to evaluate the repeatability and reproducibility of gene expression data obtained by RNA-Seq, a RT-qPCR assay using SYBR Green chemistry (Fast Start Universal Sybr green master, Rox; Roche Applied Science, Mannheim, Germany) and the comparative Ct method [62] was performed in an ABI PRISM® 7900 HT (Applied Biosystems, Inc., FosterCity, CA).

The isolated RNA of individual samples was reverse-transcribed into cDNA using the *High Capacity Reverse cDNA transcription Kit* (Applied Biosystems) in a total volume of 20 μl containing 1 μg of total RNA, following the manufacturer’s instructions. PCR primers were designed using Primer Express™ software (Applied Biosystems) and are shown in Additional file 12, Table S4. Two genes: *β-2 microglobulin* (*β2m*) and *hypoxanthine phosphoribosyltransferase 1* (*HPRT1*), previously validated as stable expressed control genes in liver tissue by geNorm [63] were used as endogenous controls (Corominas et al., unpublished data). Due to the comparative Ct method requiring the target and endogenous PCR efficiencies to be nearly equal, validation experiments for each gene were performed. Thus, the log cDNA dilution (1:2, 1:20, 1:200, 1:2,000) versus ΔCt, was plotted to obtain absolute slopes < 0.1 in all cases that allowed the use of the 2^-ΔΔCt^ method. PCR amplifications were performed in a total volume of 20 μl containing 5 μl of cDNA sample diluted 1:125. Depending on the pair primers, various concentrations were utilized (see Additional file 12, Table S4). Each sample was analyzed by triplicate, thermal cycle was: 10 min at 95°C and 40 cycles of 15 sec at 95°C and 1 min at 60°C. A dissociation curve was drawn for each primer pair. Data was analyzed using the SDS v2.4 and DataAssist^TM^ v3.0 software (Applied Biosystems). The sample of lowest expression level was selected as calibrator. Correlation between RNA-seq (Htseq) and RT-qPCR data (2^-ΔΔCt^) was carried out with R.

Copy number variation was quantified using the assay described above with some modifications. PCR amplification was carried out with 10 ng of genomic DNA isolated from diaphragm samples by the phenol-chloroform method [64]. Primers used to amplify *CYP2C49* gene are described in Additional file 12, Table S4. For single copy endogenous control gene amplification, a previously described design on the *glucagon* (*GCG*) gene [65] was used, but a single nucleotide substitution on primer forward was introduced to adapt the primer to the porcine species (Additional file 12, Table S4). A sample with the lowest copy number was selected as a calibrator.

### Transposable element analysis

To identify repetitive and transposable elements in pig liver transcriptome RepeatMasker version open-3.3.0 [http://www.repeatmasker.org/] and the ‘quick search’ and ‘pig’ species options with Search Engine: NCBI/RMBLAST and complete Database: 20090604 were used.

### Orthology and lncRNA detection

Intergenic expressed regions not yet annotated in the Sscrofa 9.61 pig genome assembly as described in [23] were extracted. Then, a conservative approach was followed, using only sequences expressed in at least four of the five animals of each group (H and L). To identify which of these transcripts were putative coding transcripts the Augustus software was used [36], providing exon boundaries and allowing complete protein translation from the forward strand. Finally, BLASTP was employed to check which of these predicted proteins were already annotated in the *Homo sapiens*, *Bos taurus* and *Sus scrofa* protein databases. For lncRNA annotation, the intergenic expressed regions were compared by BLAST with the 2,047 putative porcine lncRNA reported by Esteve-Codina et al. (2011). All transcripts that matched with an expectation value lower than 10^-5^ were retained.

### Gene functional classification, network and canonical pathways analyses

Biological network generation, functional classification and pathways analyses of differentially-expressed genes were carried out using Ingenuity Pathways Analysis software (IPA; Ingenuity Systems, http://www.ingenuity.com). The list of human homologs that correspond to the 50 protein-coding pig genes differentially-expressed was uploaded into the application. Then, each gene identifier was mapped to its corresponding gene object in the Ingenuity Pathways Knowledge Base (IPKB). Networks of these genes were generated based on their connectivity. Network analysis returns a score that ranks networks according to their degree of relevance to the network eligible molecules in the dataset [66]. The network score is based on the hypergeometric distribution and is calculated with the right-tailed Fisher’s exact test. The score is the negative log of this p*-*value. Only those molecules that demonstrate direct and indirect relationships to other genes, or proteins were integrated into the analysis.

IPA Functional Analysis was employed to identify the most significant biological functions in the comparative dataset of H and L groups. A canonical pathways analysis was generated to identify the pathways from the IPA library that were most significant. Fischer’s exact test was employed to calculate a p-value which determines the probability that each biological functions and/or canonical pathway is due to chance alone. The cut-off for considering a significance association was established by Benjamini & Hochberg (B-H) multiple testing correction of the p-value (FDR < 0.05) [67].

### Data availability

The full data sets have been submitted to Gene Expression Omnibus (GEO) under accession GSE38588 and at NCBI Sequence Read Archive (SRA) under Accession SRA053452, Bioproject: PRJNA168072.

## Abbreviations

IMF: Intramuscular fat; IPA: Ingenuity Pathways Analysis; ALA: α-linolenic acid; LA: Linolenic acid; EDA: Eicosadienoic acid; ETE: Eicosatrienoic acid; AA: Arachidonic acid; LPS: Endotoxin lipopolysaccharide; IL-1: Pro-inflammatory cytokines; RXR: Retinoid X receptors; PXR: Pregnane X receptor; FXR: Farnesoid X receptor; PPAR-α: Peroxisome proliferator-activated receptors alpha.

## Competing interests

The authors declare that they have no competing interests.

## Authors' contributions

YRC and JMF conceived and designed the experiment. JMF was the principal investigator of the project. YRC and AEC performed the RNA-Seq data analysis. YRC and NM performed the pathways analysis and drafted the manuscript. JE, AIF, MPE, NIE and JMF collected the samples. AC, MB and JC performed DNA and RNA isolation. AC, MB and JC performed the qPCR and RT-PCR assays. All authors read and approved the final manuscript.

## Supplementary Material

Additional file 1 **Table S1.** Phenotypic means comparison ± standard deviation between the sequenced individuals. Click here for file

Additional file 2 **Figure S1.** Profile of gene expression distribution in both High and Low groups. Click here for file

Additional file 3 **Figure S2.** Correlations between expression values of genes analysed by both RNA-seq and Affymetrix microarray technologies. X-axis values are the log2 of expression quantified with Affymetrix Microarray technology and y-axis are values of log2 (FPKM). Click here for file

Additional file 4 **Table S2.** Description of the repetitive elements identified in the pig liver transcriptome. Click here for file

Additional file 5 **Figure S3.** Venn diagrams of the predicted lncRNA. Click here for file

Additional file 6 **Figure S4.** Per-gene estimates of the base variance against the base levels. The red line represents the fit variance. X-axis values are the base mean and y-axis values are the log10 of the base mean and y-axis values are the log10 of the base variance. Click here for file

Additional file 7 **Figure S5.** Curves of the empirical cumulative density functions in both H and L groups. X-axis values are the chi-squared probability of residual and y-axis values are the empirical cumulative density functions. Click here for file

Additional file 8 **Figure S6.** Estimated variances as squared coefficients of variation produced with the function ‘scvPlot’. X-axis values are the base mean and y-axis values are the squared coefficients of variation. Click here for file

Additional file 9 **Table S3.** Comparison between RNA-seq (Htseq) and RT-qPCR (relative quantification) expression data of *APOA2, LPIN1, ME3, CYP7A1* and *CYP2C49* genes. Relative CNV data for CYP2C49 in comparison to the reference individual H3 is indicated in the last column. Click here for file

Additional file 10 **Figure S7.** Network 2 as generated by IPA. The significant biological functions comprising this network are Lipid Metabolism, Molecular Transport and Small Molecule Biochemistry. The network is displayed graphically as nodes (gene/gene products) and edges (the biological relationship between nodes). The node colour indicates the expression of genes: red up-regulated, green down-regulated in H group relative to L group. The shapes of nodes indicate the functional class of the gene product. Click here for file

Additional file 11 **Figure S8.** Network 3 as generated by IPA. The significant biological functions comprising this network are Carbohydrate Metabolism, Lipid Metabolism and Molecular Transport. The network is displayed graphically as nodes (gene/gene products) and edges (the biological relationship between nodes). The node colour indicates the expression of genes: red up-regulated, green down-regulated in H group relative to L group. The shapes of nodes indicate the functional class of the gene product. Click here for file

Additional file 12 **Table S4.** Primers designed for the validation of differentially-expressed genes by RT-qPCR and copy number determination by qPCR. Click here for file
