# Supplementary material for: Liver transcriptome profile in pigs with extreme phenotypes of intramuscular fatty acid composition
Source: BMC Genomics. 2012 Oct 11;13:547. doi: 10.1186/1471-2164-13-547 (PMC3478172; doi:10.1186/1471-2164-13-547)
Supplement: Additional file 1 — Table S1. Phenotypic means comparison ± standard deviation between the sequenced individuals. [file 1471-2164-13-547-S1.doc]

| **Carcass quality** | **Mean Lower** | **Mean Higher** | **Significance** |
| --- | --- | --- | --- |
| Carcass height (CH) | 68.42 ± 7.88 | 65.64 ± 11.66 | NS |
| Weight of ham (WH) | 18.56 ± 1.99 | 18.60 ± 2.54 | NS |
| Weight of Shoulder (WS) | 9.55 ± 0.73 | 9.54 ± 1.71 | NS |
| Intramuscular Fat (IMF) | 1.90 ± 0.85 | 1.93 ± 0.65 | NS |
|  |  |  |  |
| **Fatty acids** |  |  |  |
| ***Saturated FA*** |  |  |  |
| Myristic acid | 1.21 ± 0.08 | 1.09 ± 0.12 | NS |
| Palmitic acid | 23.81 ± 0.45 | 21.20 ± 0.59 | *** |
| Heptadecanoic acid | 0.19 ± 0.01 | 0.34 ± 0.06 | ** |
| Stearic acid | 14.74 ± 1.20 | 13.51 ± 1.05 | NS |
| Arachidic acid | 0.24 ± 0.06 | 0.25 ± 0.09 | NS |
|  |  |  |  |
| ***Monounsaturated FA*** |  |  |  |
| Palmitoleic acid | 2.75 ± 0.27 | 2.34 ± 0.32 | * |
| Heptadecenoic acid | 0.19 ± 0.06 | 0.33 ± 0.08 | * |
| Oleic acid | 42.78 ± 1.23 | 36.38 ± 3.28 | ** |
| Octadecenoic acid | 4.09 ± 0.17 | 3.87 ± 0.21 | NS |
| Eicosenoic acid | 0.83 ± 0.06 | 0.81 ± 0.14 | NS |
|  |  |  |  |
| ***Polyunsaturated FA*** |  |  |  |
| Linoleic acid | 6.84 ± 0.47 | 13.78 ± 1.43 | *** |
| α-Linolenic acid | 0.49 ± 0.07 | 1.19 ± 0.45 | * |
| Eicosadienoic acid | 0.41 ± 0.04 | 0.58 ± 0.16 | NS |
| Eicosatrienoic acid | 0.15 ± 0.02 | 0.45 ± 0.15 | * |
| Arachidonic acid | 0.76 ± 0.15 | 3.04 ± 1.22 | * |
|  |  |  |  |
| ***Metabolic ratios*** |  |  |  |
| Average Chain Length | 17.43 ± 0.01 | 17.51 ± 0.001 | * |
| Saturated FA | 40.21 ± 1.45 | 36.42 ± 1.19 | ** |
| Monounsaturated FA | 50.99 ± 1.70 | 44.21 ± 3.15 | ** |
| Polyunsaturated FA | 8.66 ± 0.62 | 19.07 ± 2.86 | *** |
| Peroxidability index | 12.90 ± 0.97 | 30.97 ± 6.87 | ** |
| Double-bond index | 0.70 ± 0.01 | 0.90 ± 0.05 | ** |
| Unsaturated index | 1.75 ± 0.09 | 2.47 ± 0.17 | *** |

Phenotypic means comparison ± standard deviation between the ten sequenced animals.

* p-value < 0.05, ** p-value < 0.01, *** p-value < 0.001
