# Supplementary figures and images for: Liver transcriptome profile in pigs with extreme phenotypes of intramuscular fatty acid composition
Source: BMC Genomics. 2012 Oct 11;13:547. doi: 10.1186/1471-2164-13-547 (PMC3478172; doi:10.1186/1471-2164-13-547)

High

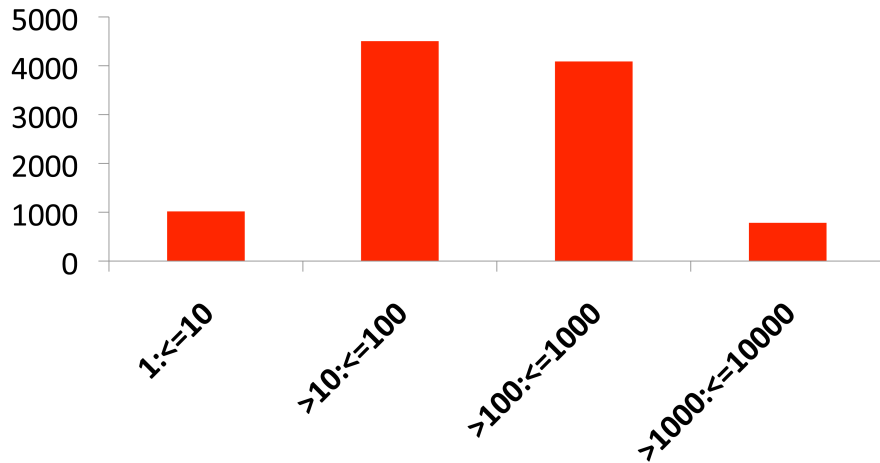

Low

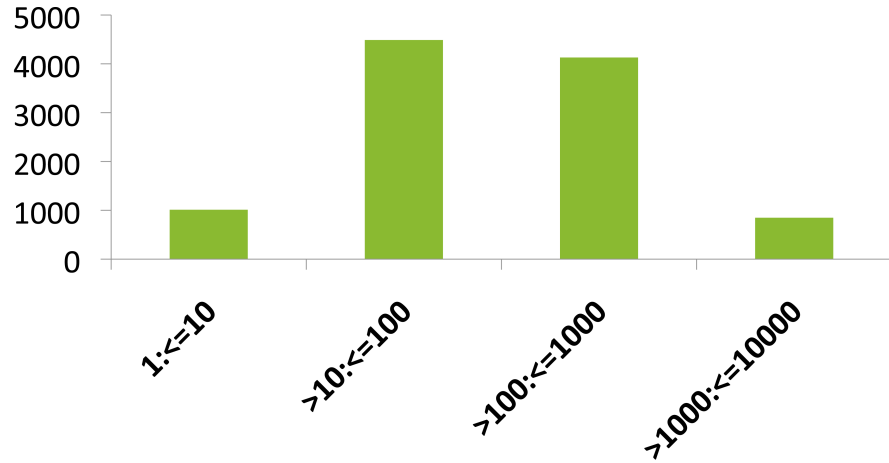

Supplement: Additional file 2 — Figure S1. Profile of gene expression distribution in both High and Low groups. [file 1471-2164-13-547-S2.pdf]

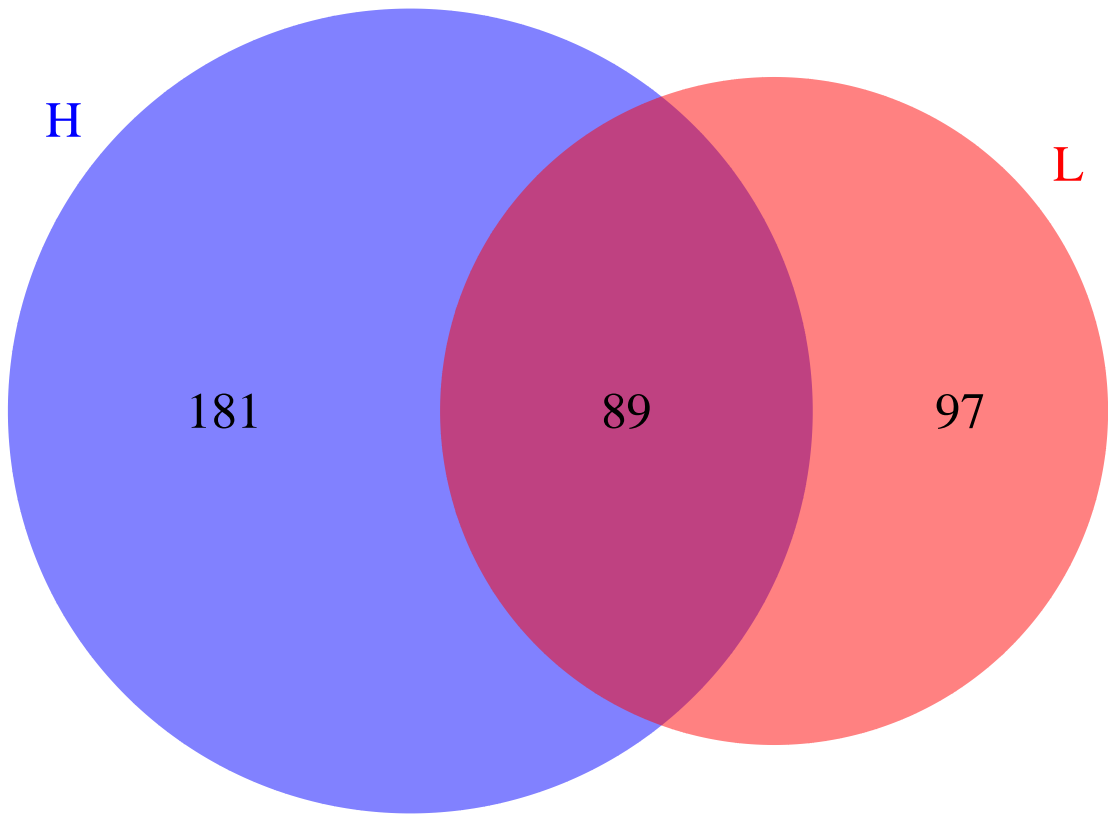

Supplement: Additional file 5 — Figure S3. Venn diagrams of the predicted lncRNA. [file 1471-2164-13-547-S5.pdf]

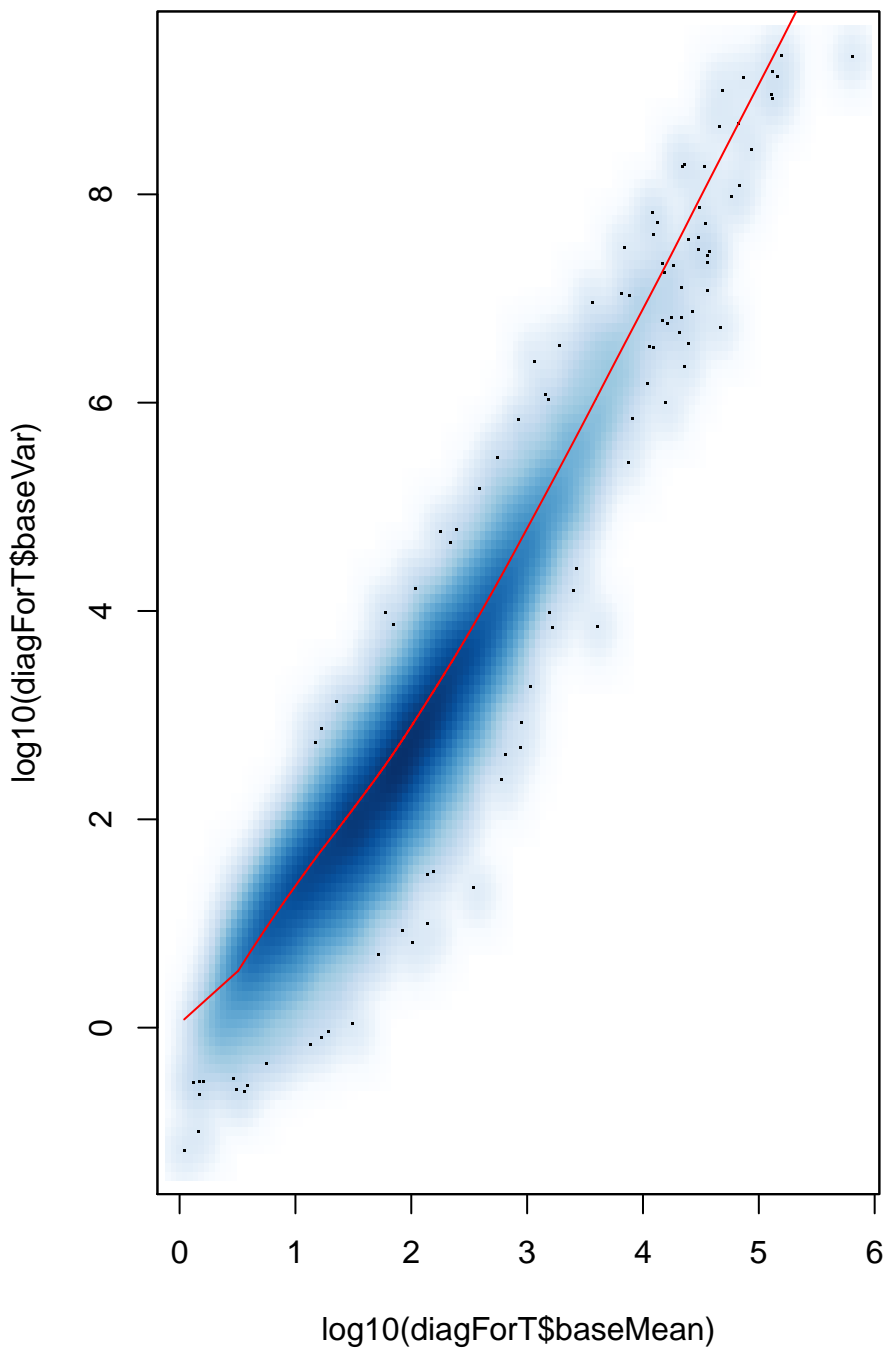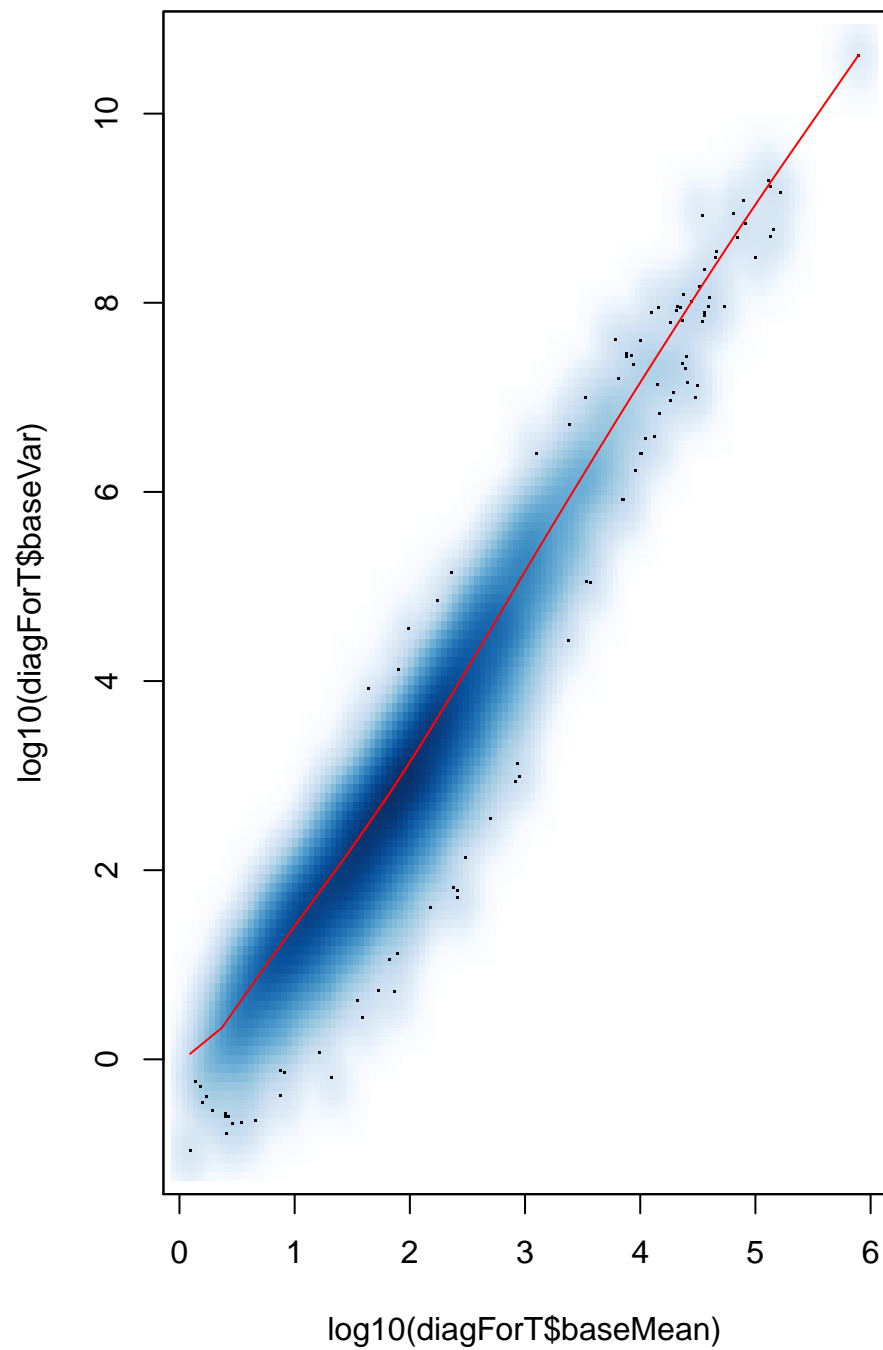

Supplement: Additional file 6 — Figure S4. Per-gene estimates of the base variance against the base levels. The red line represents the fit variance. X-axis values are the base mean and y-axis values are the log10 of the base mean and y-axis values are the log10 of the base variance. [file 1471-2164-13-547-S6.pdf]

Residuals ECDF plot for condition 'H'

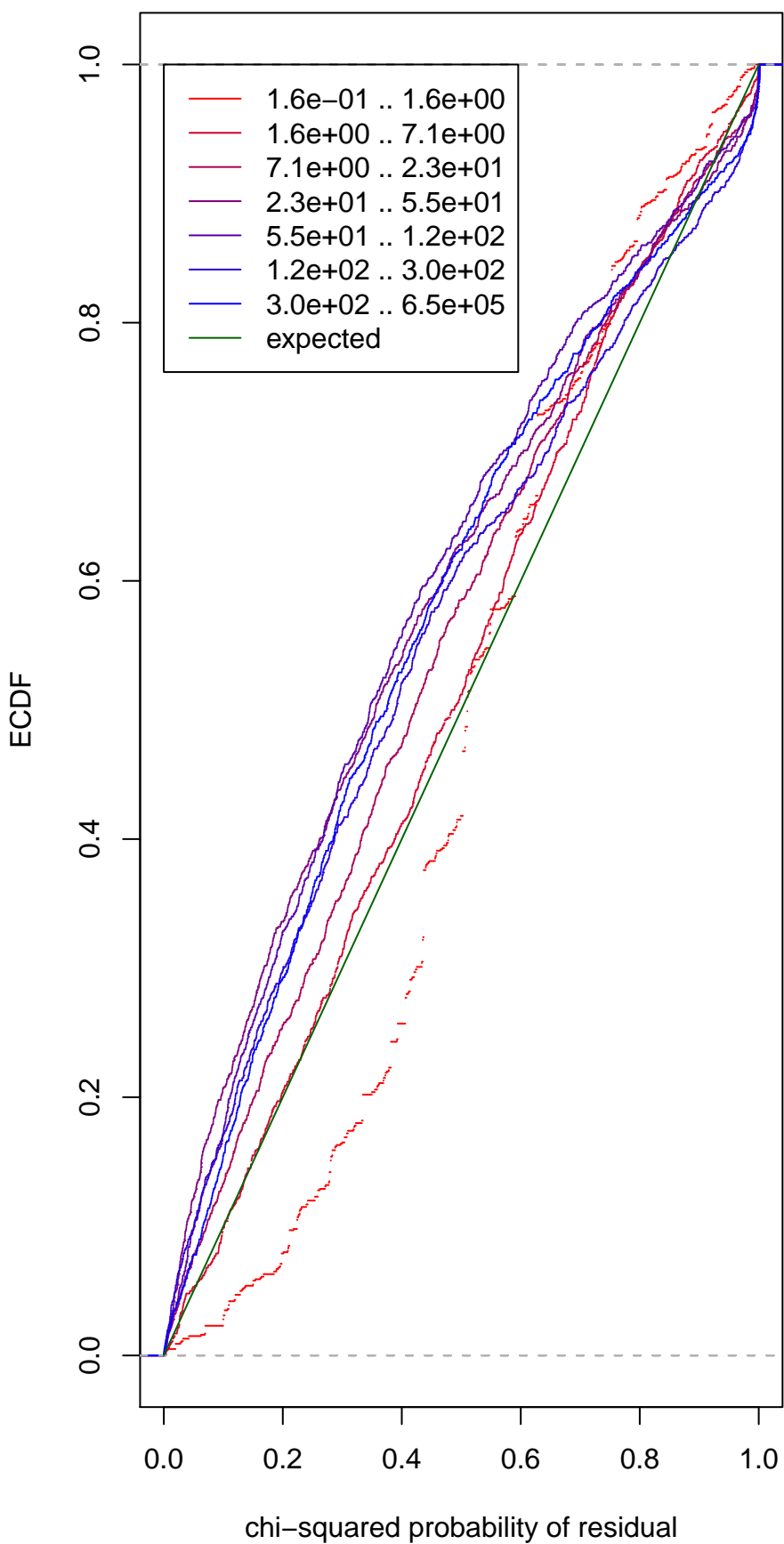

Residuals ECDF plot for condition 'L'

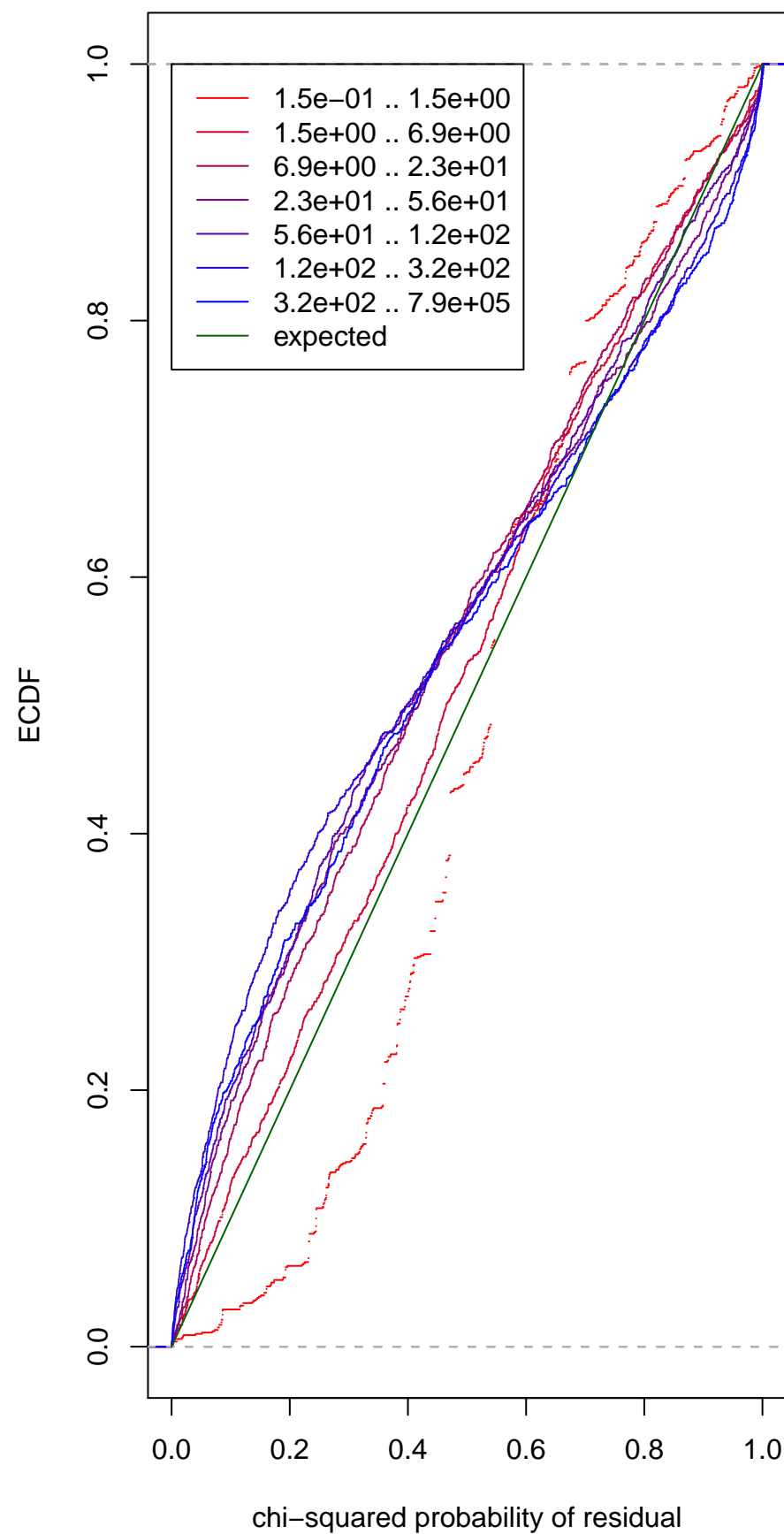

Supplement: Additional file 7 — Figure S5. Curves of the empirical cumulative density functions in both H and L groups. X-axis values are the chi-squared probability of residual and y-axis values are the empirical cumulative density functions. [file 1471-2164-13-547-S7.pdf]

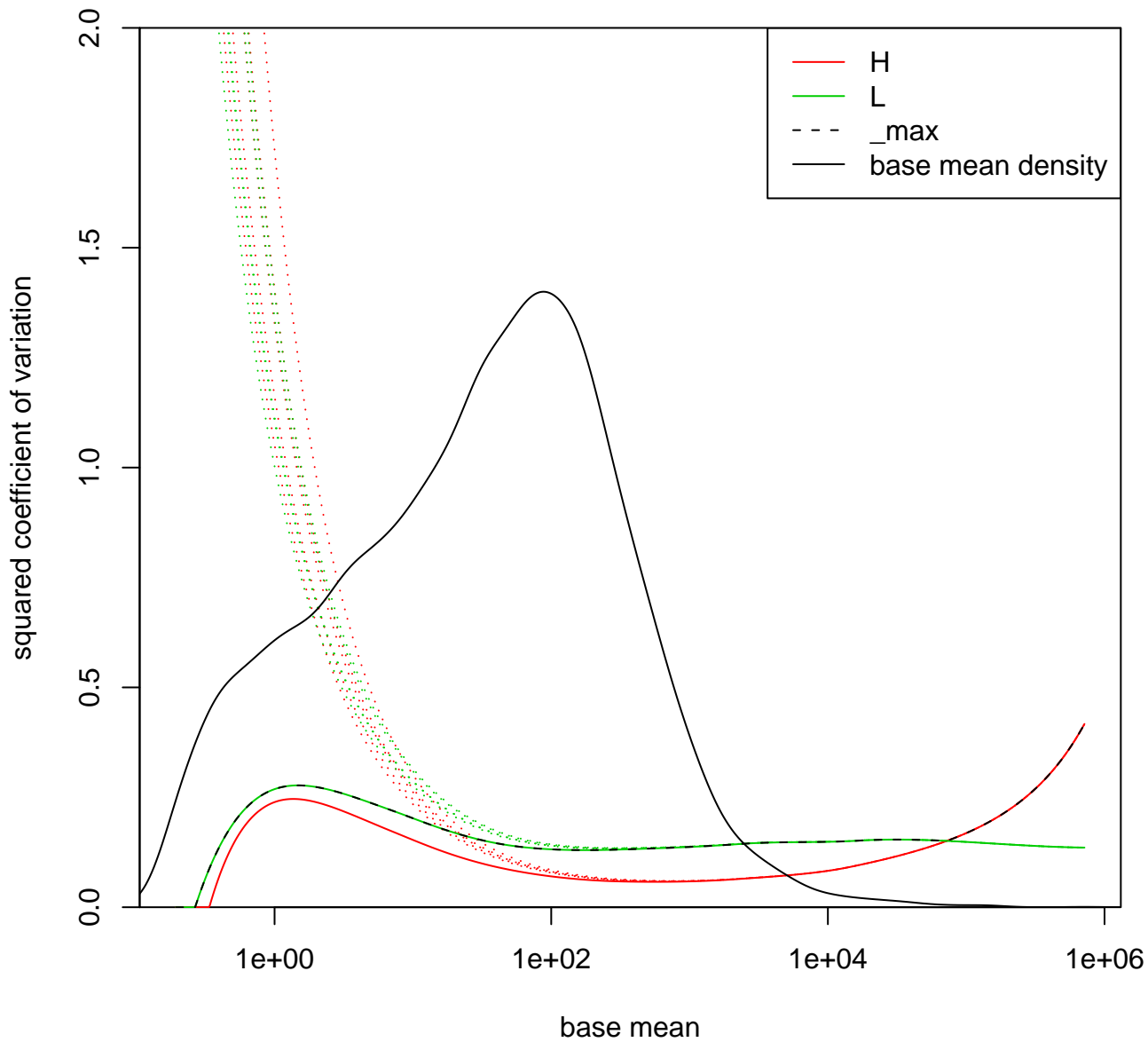

Supplement: Additional file 8 — Figure S6. Estimated variances as squared coefficients of variation produced with the function ‘scvPlot’. X-axis values are the base mean and y-axis values are the squared coefficients of variation. [file 1471-2164-13-547-S8.pdf]

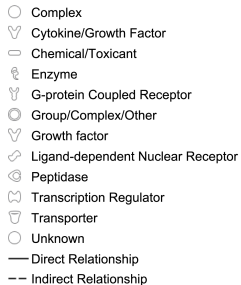

Supplement: Additional file 10 — Figure S7. Network 2 as generated by IPA. The significant biological functions comprising this network are Lipid Metabolism, Molecular Transport and Small Molecule Biochemistry. The network is displayed graphically as nodes (gene/gene products) and edges (the biological relationship between nodes). The node colour indicates the expression of genes: red up-regulated, green down-regulated in H group relative to L group. The shapes of nodes indicate the functional class of the gene product. [file 1471-2164-13-547-S10.pdf]

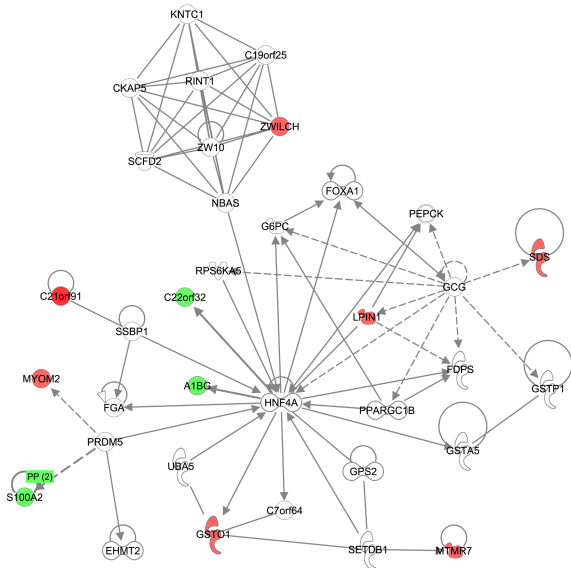

Supplement: Additional file 11 — Figure S8. Network 3 as generated by IPA. The significant biological functions comprising this network are Carbohydrate Metabolism, Lipid Metabolism and Molecular Transport. The network is displayed graphically as nodes (gene/gene products) and edges (the biological relationship between nodes). The node colour indicates the expression of genes: red up-regulated, green down-regulated in H group relative to L group. The shapes of nodes indicate the functional class of the gene product. [file 1471-2164-13-547-S11.pdf]
