# Supplementary material for: Liver transcriptome profile in pigs with extreme phenotypes of intramuscular fatty acid composition
Source: BMC Genomics. 2012 Oct 11;13:547. doi: 10.1186/1471-2164-13-547 (PMC3478172; doi:10.1186/1471-2164-13-547)
Supplement: Additional file 3 — Figure S2. Correlations between expression values of genes analysed by both RNA-seq and Affymetrix microarray technologies. X-axis values are the log2 of expression quantified with Affymetrix Microarray technology and y-axis are values of log2 (FPKM). [file 1471-2164-13-547-S3.pdf]

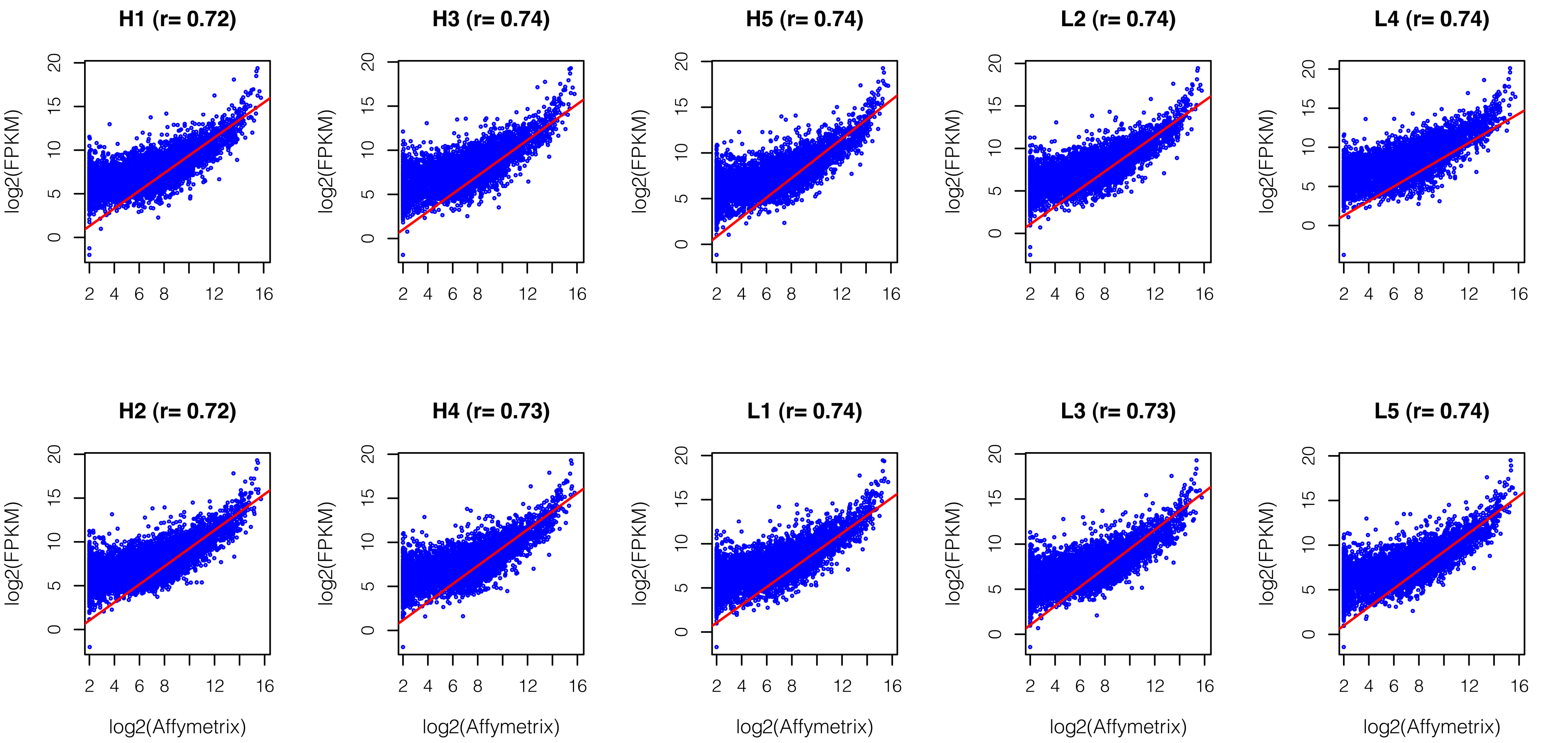
H1 (r= 0.72)
H3 (r= 0.74)
H5 (r= 0.74)
L2 (r= 0.74)
L4 (r= 0.74)
H2 (r= 0.72)
H4 (r= 0.73)
L1 (r= 0.74)
L3 (r= 0.73)
L5 (r= 0.74)
log2(FPKM)
log2(Affymetrix)
0
5
10
15
20
2
4
6
8
12
16
