# Supplementary material for: Liver transcriptome profile in pigs with extreme phenotypes of intramuscular fatty acid composition
Source: BMC Genomics. 2012 Oct 11;13:547. doi: 10.1186/1471-2164-13-547 (PMC3478172; doi:10.1186/1471-2164-13-547)
Supplement: Additional file 9 — Table S3. Comparison between RNA-seq (Htseq) and RT-qPCR (relative quantification) expression data of APOA2, LPIN1, ME3, CYP7A1 and CYP2C49 genes. Relative CNV data for CYP2C49 in comparison to the reference individual H3 is indicated in the last column. [file 1471-2164-13-547-S9.doc]

|  | ***APOA2*** | | ***LPIN1*** | | ***ME3*** | | ***CYP7A1*** | | ***CYP2C49*** | | |
| --- | --- | --- | --- | --- | --- | --- | --- | --- | --- | --- | --- |
| Sample name | RNA-seq | RT-qPCR | RNA-seq | RT-qPCR | RNA-seq | RT-qPCR | RNA-seq | RT-qPCR | RNA-seq | RT-qPCR | CNV |
| L1 | 841 | 1.44 | 103 | 2.63 | 17 | 1.03 | 11 | 4.74 | 3514 | 10.5 | 3.1 |
| L2 | 710 | 1.13 | 179 | 1.99 | 31 | 1.01 | 237 | 75.03 | 817 | 1.9 | 4.7 |
| L3 | 616 | 1.00 | 33 | 1.00 | 34 | 1.05 | 11 | 2.02 | 817 | 1.0 | 2.4 |
| L4 | 1377 | 2.79 | 56 | 1.46 | 139 | 3.40 | 15 | 2.89 | 999 | 1.3 | 1.3 |
| L5 | 1307 | 2.50 | 103 | 2.05 | 88 | 1.95 | 295 | 39.02 | 1767 | 1.1 | 2.1 |
| H1 | 981 | 1.99 | 163 | 1.98 | 18 | 1.00 | 1068 | 141.84 | 2333 | 3.4 | 5.2 |
| H2 | 2508 | 3.94 | 214 | 4.29 | 27 | 1.54 | 69 | 17.07 | 6793 | 14.3 | 2.0 |
| H3 | 1456 | 2.29 | 435 | 5.58 | 44 | 1.37 | 154 | 56.98 | 6086 | 10.4 | 1.0 |
| H4 | 3534 | 3.07 | 20 | 1.48 | 24 | 1.10 | 4 | 1.00 | 7334 | 7.3 | 2.0 |
| H5 | 964 | 2.28 | 123 | 1.35 | 46 | 1.59 | 71 | 11.68 | 1884 | 3.5 | 4.6 |
| Correlation | 0.79 | | 0.90 | | 0.96 | | 0.93 | | 0.85 | |  |

Comparison between RNA-seq (Htseq) and RT-qPCR (relative quantification) expression data of *APOA2*, *LPIN1*, *ME3*, *CYP7A1* and *CYP2C49* genes. CNV data for *CYP2C49* is indicated in the last column.
