# Supplementary material for: Liver transcriptome profile in pigs with extreme phenotypes of intramuscular fatty acid composition
Source: BMC Genomics. 2012 Oct 11;13:547. doi: 10.1186/1471-2164-13-547 (PMC3478172; doi:10.1186/1471-2164-13-547)
Supplement: Additional file 12 — Table S4. Primers designed for the validation of differentially-expressed genes by RT-qPCR and copy number determination by qPCR. [file 1471-2164-13-547-S12.docx]

|  | **Name** | **Sequence (5’-3’)** | **[Primer]** | **Amplicon lenght (bp)** |
| --- | --- | --- | --- | --- |
| **CNV detection** | CYP 2C49-DNA-F | GCATGGAGCTATTTTTATTCCTGACT | 300 nM | 111 |
|  | CYP2C49-R | TATGGCTGAACCCACTTAAAATG | 300 nM |  |
|  | GCG-F | AACATTGCCAAACGTCACGATG | 600 nM | 130 |
|  | GCG-R | GCCTTCCTCGGCCTTTCA | 600 nM |  |
| **RNA-Seq validation** | CYP7A1-RNA-F | TGACGCAGAGAAAGCCAAGTC | 900 nM | 127 |
|  | CYP7A1-RNA-R | TTCAGAAGCTGCTTTCATTGCT | 900 nM |  |
|  | CYP2C49-RNA-F | TCTCAACAGGAAAACGAATTTGTG | 300 nM | 153 |
|  | CYP2C49-R | TATGGCTGAACCCACTTAAAATG | 300 nM |  |
|  | LPIN1-F | CCGAGAGAAGGTGGTGGACAT | 600 nM | 76 |
|  | LPIN1- R | CTCTCCATTGTCTCCCAGTTTCA | 600 nM |  |
|  | ME3-F | TGACCTGGACAAGTACATCATTCTG | 600 nM | 72 |
|  | ME3 -R | GTCAGCACTCGGTAGAAGAGCTT | 600 nM |  |
|  | APOA2-F | AACTTCAAACTCAGGCCAAGACTTA | 300 nM | 111 |
|  | APOA2-R | GGCCAATGAAACTGCTCAAGA | 300 nM |  |
|  | β2M RT-F | ACCTTCTGGTCCACACTGAGTTC | 600 nM | 108 |
|  | β2M RT-R | GGTCTCGATCCCACTTAACTATCTTG | 600 nM |  |
|  | HPRT1 RT-F | TCATTATGCCGAGGATTTGGA | 900 nM | 91 |
|  | HPRT1 RT-R | CTCTTTCATCACATCTCGAGCAA | 900 nM |  |

**Additionally File 10 Table 3**. Primers designed for the validation of differential expressed genes by RT-qPCR and copy number determination by qPCR
